# Incorporating an intersectional gender approach to improve access to maternal and child health screening services

**DOI:** 10.1186/s12939-024-02109-3

**Published:** 2024-02-20

**Authors:** Margarita Rivera Arrivillaga, Marina Gold, Elizabeth Pellecer Rivera, Jose Guillermo Juárez

**Affiliations:** 1 Centro de Estudios en Salud, Universidad del Valle de Guatemala, 18 Av. 11-95 Zona 15 VH III, Guatemala City, Guatemala; 2 Fundación Mundo Sano, Recaredo, 3. Puerta Garaje, 28002 Madrid, Spain; 3 Environmental Sciences, University of Maine, Orono, ME 04469-5755 USA

**Keywords:** Guatemala, Midwives, Chagas, Syphilis, Hepatitis B, HIV, Intersectionality, EMTCT diseases

## Abstract

**Background:**

In the Americas, the Pan American Health Organization (PAHO) has promoted initiatives that aim at the elimination of mother-to-child transmitted diseases for over two decades. Although Guatemala has assumed the commitment to improve access and coverage of reproductive and perinatal services, the goals have not yet been reached. Often, the implementation of these efforts is hampered by complexities rooted in social, cultural, and environmental intersections. The objective of this work is to share our experience applying gender intersectionality as a methodological and analytical tool in a participatory research project that aims to improve access to maternal and child health screening services. The study shows the novel strategy that incorporates intersectionality contributing to evidence on how it can be applied to strengthen public health efforts around the implementation of the EMTCT Plus (Elimination of mother-to-child transmission of HIV, Syphilis, Hepatitis B, and Chagas disease) framework, in the mostly rural municipality of Comapa, in Guatemala.

**Methods:**

We applied a participatory methodology, integrating theoretical and methodological frameworks to have an intersectional understanding of health services delivered by both, midwives, and the public health institution, for the prevention, diagnosis, treatment, and follow-up of HIV, Syphilis, Hepatitis B, and Chagas. The data was collected by conducting interviews, focus groups, workshops, and reviewing laboratory databases, guided by five strategies from a cultural appropriateness framework.

**Results:**

The intersectional analysis shed light on the synergies and gaps of the current efforts and protocols implemented by both the midwives and the Ministry of Health. The services offered for the four diseases were often delivered independently from each other, and a comprehensive educational and communication material strategy was absent. However, our findings will be used to inform consistent, locally relevant, and culturally appropriate educational content for the local population, also following the national policy guidelines.

**Conclusions:**

Using intersectionality as a method and as an analytical tool allowed us to understand the (1) interrelation of diverse social, cultural, and environmental determinants which influence the delivery of health services, as well as (2) the dynamics between the traditional and institutional health systems. (3) Community engagement and the participation of different stakeholders in a consultative process have been fundamental for the conceptual and methodological tenets of this research. (4) Finally, giving a more prominent role to midwives can strengthen sustainability and cultural appropriateness, which is complementary to the delivery of institutional health services.

**Supplementary Information:**

The online version contains supplementary material available at 10.1186/s12939-024-02109-3.

## Resumen

**Antecedentes:**

La Organización Panamericana de la Salud (OPS) ha impulsado iniciativas que buscan la eliminación de las enfermedades de transmisión materno infantil en las Américas desde hace más de dos décadas. Si bien Guatemala ha asumido el compromiso de mejorar el acceso y la cobertura de los servicios reproductivos y perinatales, las metas aún no se han alcanzado. Muchas veces, la implementación de estos esfuerzos se ve obstaculizada por complejidades arraigadas en intersecciones sociales, culturales y ambientales. Este estudio muestra una estrategia novedosa que incorpora la interseccionalidad como un componente metodológico y analítico. Esto contribuye a evidenciar la manera en que la interseccionalidad y la participación comunitaria pueden ser aplicadas para fortalecer los esfuerzos de salud pública en torno a la implementación de la estrategia ETMI Plus (Eliminación de la transmisión materno infantil del VIH, sífilis, hepatitis B y enfermedad de Chagas), en el municipio de Comapa, en Guatemala, el cual es mayoritariamente rural.

**Métodos:**

Implementamos una metodología participativa, integrando marcos teóricos y metodológicos para comprender la prestación de servicios de salud, tanto por parte de comadronas como de la institución de salud pública, desde una perspectiva interseccional para la prevención, diagnóstico, tratamiento y seguimiento de VIH, sífilis, hepatitis B y Chagas. Los datos fueron recolectados a través de entrevistas, grupos focales, talleres y tras la revisión de bases de datos de laboratorio, y nos guiamos por cinco estrategias propuestas en un marco para pertinencia cultural.

**Resultados:**

El análisis interseccional permitió entender las sinergias y brechas de los esfuerzos y los protocolos que se implementan actualmente, tanto por parte de las comadronas como por el Ministerio de Salud. Encontramos que los servicios que actualmente se prestan para las cuatro enfermedades son en su mayoría independientes entre sí, y no se contaba con una estrategia integral de material educativo y de comunicación. Sin embargo, nuestros resultados se utilizarán como base para una estrategia de comunicación que sea coherente, localmente relevante y culturalmente apropiada para la población local, y también siga las regulaciones de las políticas nacionales.

**Conclusiones:**

El uso de la interseccionalidad como método y como herramienta analítica nos permitió comprender (1) la interrelación de diversos determinantes sociales, culturales y ambientales que influyen en la prestación de servicios de salud, así como (2) la dinámica entre los sistemas de salud tradicional e institucional. (3) El compromiso de la comunidad y la participación de las diferentes partes interesadas en un proceso consultivo han sido fundamentales para los principios conceptuales y metodológicos de esta investigación. (4) Por último, otorgar un papel más destacado a las matronas puede reforzar la sostenibilidad y la adecuación cultural, que es complementaria a la prestación de servicios de salud institucionales.

## Background

For the past two decades, the Pan American Health Organization (PAHO) has promoted actions toward eliminating mother-to-child transmitted diseases in the Americas, with an initial focus on HIV (human immunodeficiency virus) and syphilis. In 2016, two additional diseases were included – Hepatitis B and Chagas – as described in the “Framework for the Elimination of Mother-to-Child Transmission of HIV, Syphilis, Hepatitis B, and Chagas” or EMTCT Plus. Chagas disease although mainly found in Latin America, with 70 million people at risk of infection and causing 546,000 disability-adjusted life years (DALYs) [1, 2], can now also be found in the northern hemisphere due to migration. However, since the vector is not found in many northern countries [3] the main concern is transfusion and maternal-infant care since seroprevalence of individuals from Chagas disease endemic countries can be as high as 18% [4]. In Guatemala, there are still some areas with high disease transmission with seroprevalence in women of child-bearing age of 10.5% [5]. In this sense, Guatemala as a member state of PAHO, has assumed the commitment to improving coverage and access to family planning services for women of reproductive age, including antenatal, delivery, and postnatal care, screening, vaccination, and treatment programs for EMTCT [6]. In 2019, the Guatemalan National STD (sexually transmitted diseases), HIV, and AIDS (acquired immunodeficiency syndrome) Program reported that HIV, syphilis, and hepatitis B tests were performed on pregnant women with at least one prenatal check-up. The tests performed in the country indicated that 0.82% of women tested positive for HIV, 0.21% for syphilis, and 0.19% for hepatitis B [7].

National policies in Guatemala have not yet reached the goals set out by the PAHO [8]. We argue that local initiatives that draw on community engagement are essential in the implementation of EMTCT Plus guidelines through a culturally appropriate approach. In rural and ethnically diverse regions of Guatemala, where the healthcare system is often underfunded, local midwives are key actors in providing maternal and child healthcare at the community level [9, 10]. The contribution of midwives to the national health system is officially recognized and incorporated in formal guidelines implemented by the Ministry of Health (MoH) [11], where midwives are increasingly acknowledged as important actors and stakeholders in the delivery of overall health care, going beyond women’s and children’s health. Midwives have the potential to be relevant actors in the EMTCT Plus objectives, particularly in the role they play in detecting, treating, and monitoring HIV, syphilis, hepatitis B, and Chagas patients. Overall, the role of midwives provides culturally appropriate and sustainable points of articulation between the healthcare system and the community, streamlining the implementation of screening and treatment programs, especially in rural and remote areas [7]. However, midwives – particularly those articulating different knowledge systems – tend to be excluded and marginalized from the mainstream healthcare system [12]. We take midwives as the central point of our analysis to consider how intersectionality is crucial in the development of culturally targeted and tailored programs that consider community engagement from their inception.

In 2019, our interdisciplinary team conducted a research project to strengthen the EMTCT Plus framework in Guatemala in the municipality of Comapa, Jutiapa, a context where perinatal care, gender inequalities, and power structures emerge as interrelated social determinants of health [13]. The project has consisted of subsequent phases necessary to conduct a contextually tailored program, designed with an intersectional approach, that will culminate in the production of culturally appropriate educational and communicational material for the promotion of screening and treatment of EMTCT Plus diseases [14]. The objective of this project is to incorporate an intersectional gender approach in the relationship between the local health care system and midwives, through a collaborative process of development of educational material on vertically transmitted diseases. The desired result is to strengthen the public health care system by reinforcing preventative health practices regarding mother-to-child transmitted diseases.

### Theoretical framework

Social determinants of health (SDH) are defined by the World Health Organization as “nonmedical factors that influence health outcomes” [15] and include a wide range of social and system-related factors that create conditions in which populations or individuals are born, grow, live, and work. SDH can be linked to individual, social, and structural conditions [13] and have a direct influence on inequalities produced in access to healthcare and on disease outcomes for different populations and individuals.

**Fig. 1:**
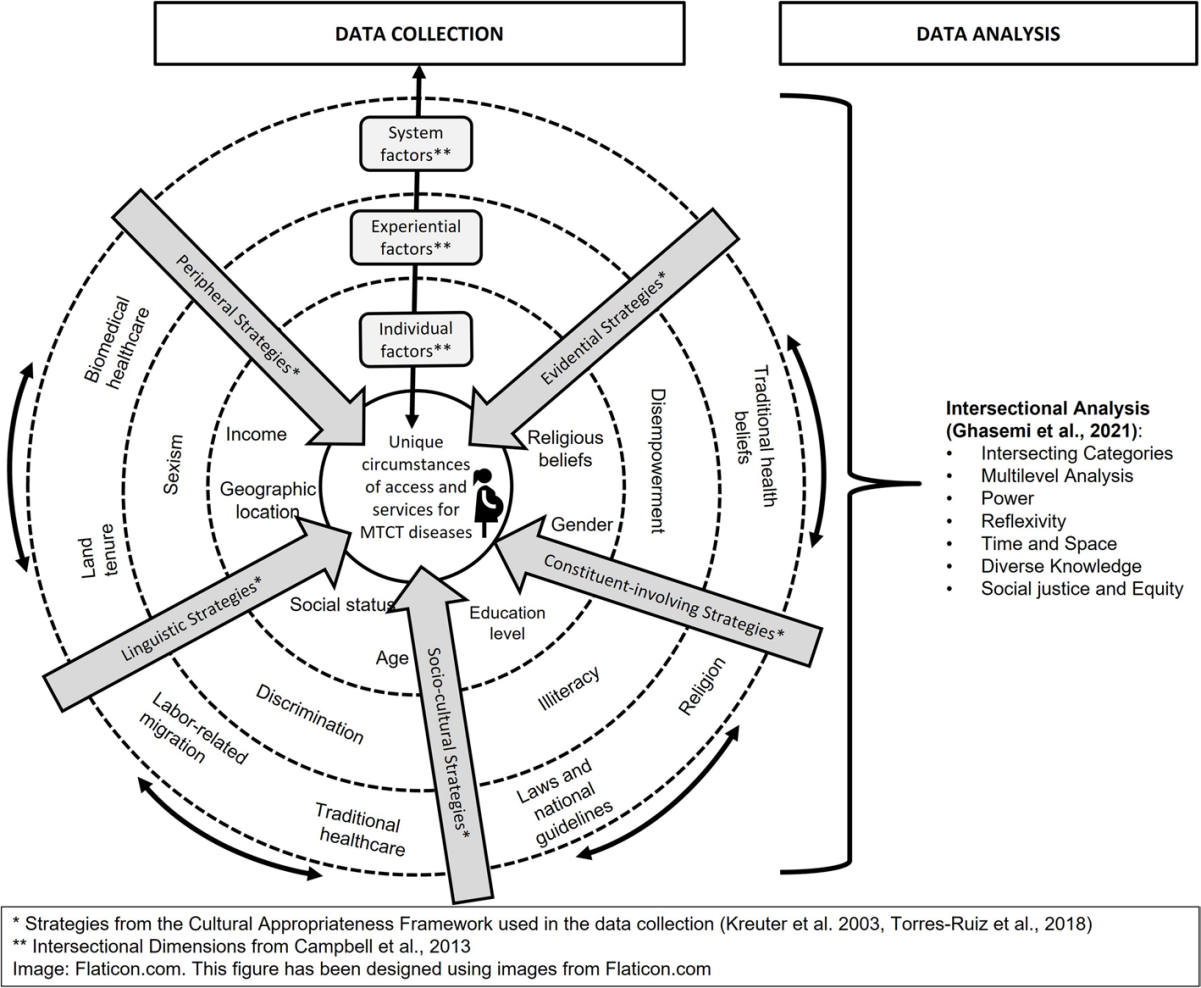
Person-Centered Theoretical Framework [17–20]

Research related to EMTCT Plus has considered SDH, such as the socioeconomic and educational conditions of individuals and populations and their access to affordable and decent health services [13, 16]. Maternal and child health are influenced by socially constructed norms, “roles, behaviors, activities, attributes, and opportunities connected to gender” [13]. Therefore, gender should be a central SDH for prevention, screening, and access to treatment of mother-to-child transmitted diseases, as inequalities and power relations generated by social and structural gender norms impact all levels of disease management. In addition, we consider other SDHs, such as socioeconomic inequalities and discrimination, institutional policies, and conditions that can influence the achievement of the EMTCT Plus goals and targets [13, 16]. Our research project has considered SDH throughout the process of design and implementation, applying an intersectional strategy with a focus on gender (Fig. 1), for which we followed a combination of Kreuter et al. (2003), Torres-Ruiz et al. (2018), Campbell et al. (2013) and Ghasemi et al.’s (2021) approaches [17–20].

Intersectionality can be understood as a method, a heuristic, and an analytical tool [21, 22] that helps to understand “how different social stratifiers interact to create different experiences of privilege, vulnerability, and/or marginalization” [13]. Intersectionality has served to address issues related to law, feminism, antiracist theory, and politics. However, it is increasingly common for scholars and activists to use this concept to approach a wide variety of issues that relate to other social identities, power dynamics, and legal and political systems. The intrinsic multidimensional characteristic of intersectionality has motivated its application across disciplines [23]. Intersectionality analysis complements the concept of SDH by focusing on “how multiple social identities at the individual level of experience (i.e., the microlevel) intersect with multiple-level social inequalities at the macro structural level” [24]. Some previous studies have applied gender intersectionality and health to understand how to tackle differences in access to affordable and comprehensive health care [16, 25], understanding the power dynamics and, the role of multiple actors involved in maternal-child healthcare [20, 26]. Intersectionality as a methodology has also been implemented to understand the connection between cultural tailoring and patient engagement to improve outcomes for populations at risk for disparities [19].

## Materials and methods

This research project has resulted from a collaboration between the Guatemalan Ministry of Health (MoH), the Universidad del Valle de Guatemala, and the international NGO Fundación Mundo Sano. The multidisciplinary research team – consisting of anthropologists, biologists, public health specialists, doctors, epidemiologists, and other healthcare personnel from the study area (i.e., nurses, vector control staff, midwives) – allowed for a comprehensive understanding of the problem of vertically transmitted diseases.

### Study site

**Fig. 2:**
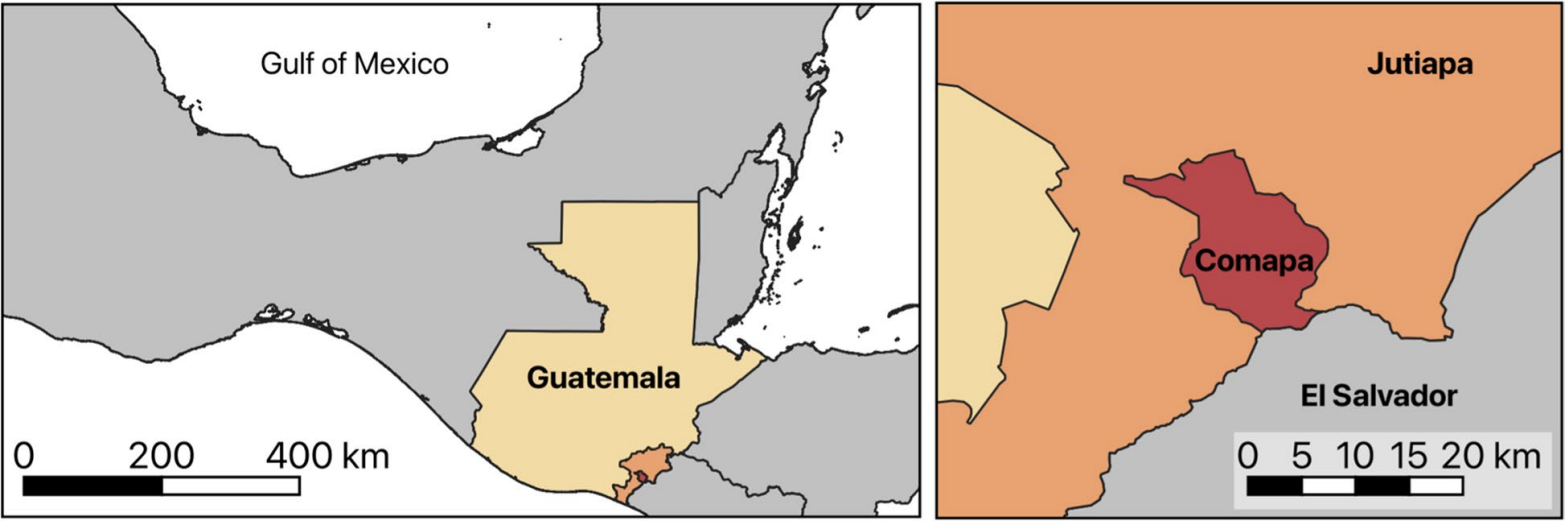
The geographic location of Comapa, Jutiapa, Guatemala. Map developed in QGIS 3.26

This work was done in Comapa, a municipality in the department of Jutiapa, located in the southeast of Guatemala (Fig. 2). The 2018 census registered 32,207 inhabitants [27]. Due to its border with El Salvador, trade and migration are key processes impacting the socioeconomic dynamics in Comapa. Geographical context also impacts health-seeking behavior, as some people prefer to seek medical treatment in El Salvador, given the conditions of the roads and proximity to local health care centers. Long-standing work by several national and international institutions in Comapa has sought the comprehensive management of Chagas disease, which continues to have infestation levels of 15% despite decades of efforts to control and eliminate vectorial transmission from this area [5, 28–30]. However, progress achieved to date in the control of vector transmission [31] has allowed us to expand our work and focus on congenital transmission.

### Study population

The municipality of Comapa is different from the Mayan Highlands on the western side of the country, where an important proportion of the population self-identifies as indigenous. Comapa is, however, ethnically diverse: 0.7% Maya, 0.2% Garifuna, 11.3% Xinka, 0.7% Afro-descendants, 87.1% Ladino, and less than 0.1% foreigners [27]. This often implies multiple knowledge systems, plurilingualism, and tense sociopolitical relations surrounding land rights. Relations within the community and between the community and the health care system are influenced by this complex ethnic configuration. This project is built on previous participatory work conducted in the area since 2015 by a multidisciplinary team from the aforementioned university that focused on strengthening Chagas congenital disease screening [14]. Community engagement – a core principle of such research – has fostered strong rapport with local midwives, MoH health personnel, and other organizations such as the international NGOs that work in Comapa.

### Data collection

**Fig. 3:**
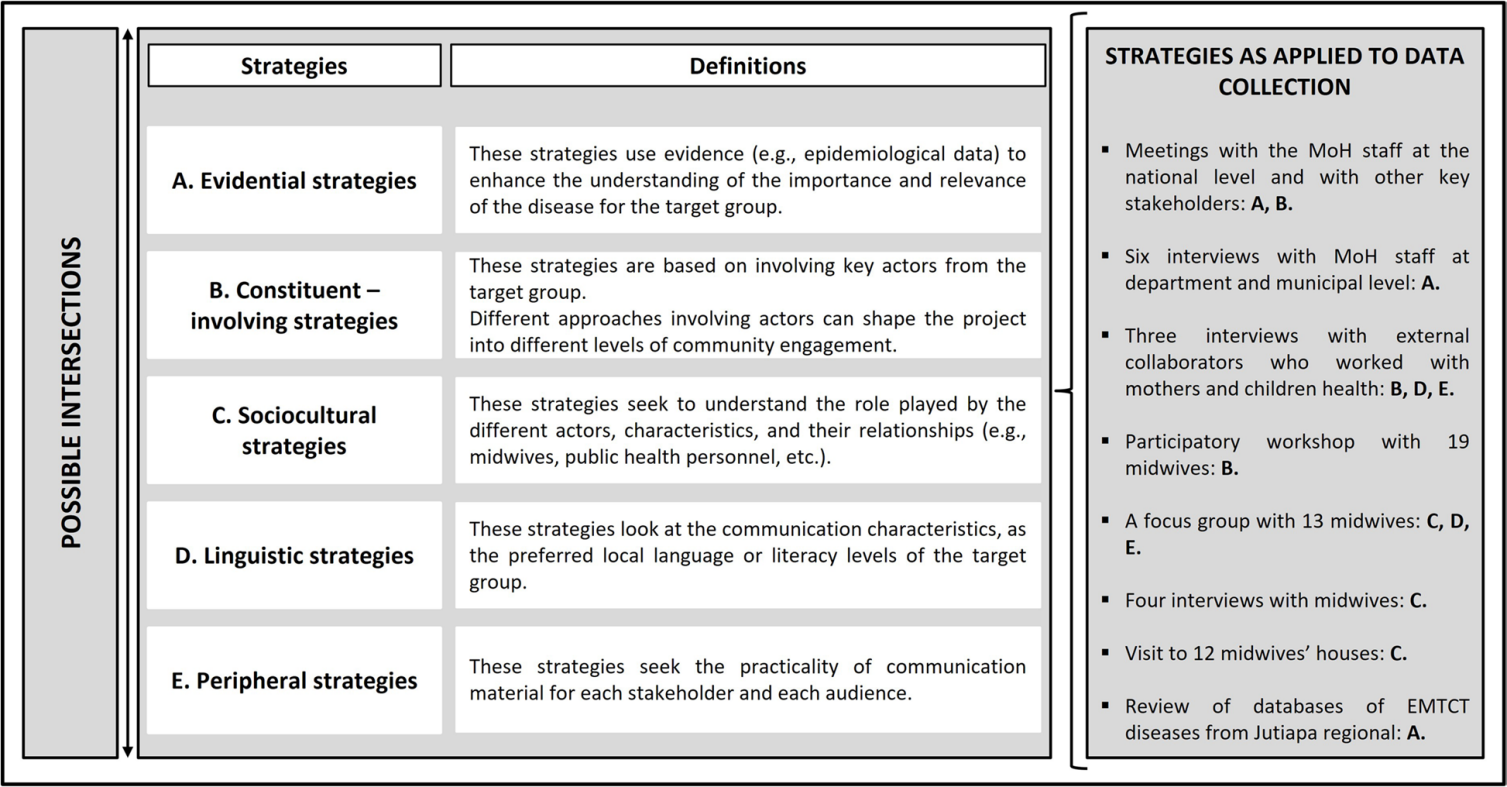
Strategies of the cultural appropriateness approach [17, 19]

We used diverse data collection methods in 2019, with the participation of midwives, staff from the MoH at both the national and local levels, and external collaborators (i.e., actors from other institutions working on topics related to maternal and children’s health in Comapa). Additionally, we reviewed official databases with screening records. The data collection process was based on five strategies from the cultural appropriateness approach: A) evidential, B) constituent-involving, C) sociocultural, D) linguistic, and E) peripheral, following Kreuter et al. (2003) and Torres-Ruiz et al. (2018) (Fig. 3) [17, 19]. The adoption of these strategies was intended to address complexities that arise in public health strategies regarding maternal and child needs in this rural context, enhancing the success and sustainability of health interventions at the community level [17].

Given that we used several data collection methods and that the five strategies from the cultural appropriateness framework are not mutually exclusive, we first provide details on the selection of the population to later explain how the strategies were applied during data collection (Fig. 3). The midwives involved with interviews and participatory activities were selected using a purposive sampling strategy based on the criteria of high attendance to the training workshops provided and required by the MoH. Additionally, we used chain referral to accompany a subset of twelve midwives to their homes for participant observation [32, 33]. We also used a purposive strategy to interview the MoH staff and the ‘external collaborators’, based on criteria sampling, targeting personnel who had collaborated with the university project previously and have a key role related to the implementation of the EMTCT Plus strategies. This group included nurses, doctors, vector control personnel, and staff from an NGO. The personnel from the Chagas vector control program have access to people’s homes and know the community, making them important partners in the region [32, 33].

The interviews and focus groups were recorded. Audio recordings were managed by the anthropologists, who listened to the recordings to make observations promptly and deepen the interviews and the information to be collected [32]. With the qualitative data obtained in the individual and group interviews, we aimed for a theoretical saturation point [32]. All midwives and nurses at the local health care center were female, and the research team from the university was mostly composed of female researchers. The international NGO team consisted of two men and one woman. The interviews and focus groups were conducted by two female anthropologists to reduce the hierarchical gender dynamics that can emerge between males and females, who additionally belong to different sociocultural groups.

#### Evidential strategies

We considered data containing screening information about patients who had been tested for any of the EMTCT Plus diseases at the Comapa local health care center and whose tests had been sent for confirmation to the laboratory at Jutiapa between 2012 and 2019. Datasets were kept in the laboratory of Jutiapa with the births registered in each municipality, including Comapa, and presented unidentifiable data of patients who had been tested for HIV, syphilis, hepatitis B, and Chagas. Information was disaggregated by sex, age, community of origin, place where the test was performed, date, and result. This information provided a snapshot of how many EMTCT Plus-related tests are carried out at the Comapa local health care center and provides a general idea of the incidence of these diseases in the municipality (Table 2).

Complementarily, to identify the prevention, diagnosis, and treatment routes of EMTCT Plus diseases in Comapa, we conducted six interviews with public health staff at the department and municipal level (Jutiapa Hospital: 2; Comapa local health care center: 4).

#### Constituent-involving strategies

We considered the involvement of stakeholders in the macro structures and community structures concerning EMTCT Plus; and the needs and expectations of local stakeholders working with mother and child healthcare, including midwives. To identify the macro structures and community structures relevant to the diagnosis and treatment of EMTCT Plus, we conducted three additional interviews with ‘external collaborators’, which were identified, as they already work with maternal and/or infant health in the area, and they could support the sustainability and acceptability of our efforts (see Fig. 3).

The needs and expectations of local stakeholders provided crucial material to develop a robust community engagement approach. A participatory workshop was held at the local health care center in Comapa, where nineteen midwives discussed the congenital transmission of HIV, syphilis, hepatitis B, and Chagas disease. The objectives of the workshop were to generate awareness about vertically transmitted diseases among midwives and to better understand perceptions of health and disease among midwives. This workshop also provided a dialog space to learn about the needs and expectations related to vertically transmitted diseases. This last activity was also considered part of the sociocultural, linguistic, and peripheral strategies.

#### Sociocultural strategies

Through participant observation, twelve midwives’ houses were visited multiple times by the team of anthropologists, aiming to better understand and complement the information generated with the other methods. These observations were contextualized within prior work conducted, as our research team has a long-standing presence in the region [14, 34]. Although we aimed to accompany midwives in their consultations with pregnant women, this was not possible due to time and resource constraints. However, when midwives visit their patients, they usually remind women of their appointments and check their well-being, which is part of their routine care.

Four in-depth interviews were conducted with selected midwives in their homes, a space where they could feel comfortable sharing their experiences. These interviews were carried out in Spanish by a woman anthropologist. The objective of these semistructured interviews was to understand how midwives perceive health and illness and their role within the healthcare system. Additionally, to complement the information gathered in the interviews, we conducted a focus group with thirteen midwives in the local health care center in one of the routine training meetings. Finally, we conducted three interviews with external collaborators in other relevant institutions identified in the previous phase as closely involved with the community and working with mothers and children.

#### Linguistic and peripheral strategies

The participants’ primary language was Spanish, and the researchers were native Spanish speakers and had conducted fieldwork in the region, understanding the subtleties of the language. Most participants had a very basic understanding of reading and writing – some could only sign their names. This meant that all interviews, communications, and focus groups had to avoid technical terms and allow for multiple repetitions of concepts. From the data generated in the participatory workshop activity, including the drawings reflecting the midwives’ understanding of key concepts, the interdisciplinary team is developing educational and communication material that reflects the community’s perspective (an emic view) on EMTCT disease prevention.

One of the main concerns of the research process has been to ensure the further acceptability of the communication strategy [14]. Finally, the interviews held with external collaborators helped us triangulate the information to identify if all institutions working with maternal health are using the same messages and linguistic codes.

### Data analysis

The complex sociocultural analysis carried out for the development of this research project can be disaggregated into categories proposed by Ghasemi and collaborators (2021) in their scoping review on intersectionality applied to health interventions: intersecting categories, multilevel analysis, power relations, reflexivity, time and space of the implementation, diverse knowledge and social justice and equity (Table 1) [18]. Within these general categories, the authors list multiple guiding questions designed to serve as general prompts to ensure the intersectionality of a program at the identification, design/implementation, and evaluation stages. We brought together the data collected from all the sources, reflecting on each of the categories and using the questions to guide the analysis. The interviews and focus group activities with midwives were categorized into four overarching themes: (1) health care center/system, (2) the social role of midwives, (3) nature, and (4) health and illness. Furthermore, other forms of data, such as stakeholder maps, epidemiological data, and literature reviews, can also be organized into these categories, which often overlap. Therefore, it is crucial to understand the interaction between the different categories to appreciate the full complexity of the situation. In this paper, we focus on the first two stages, as the third stage of evaluation has yet to be completed.

**Table 1:** Categories for Intersectional Analysis with some questions applied in our analysis process. Adapted from Ghasemi et al. (2021) [18]

Category	Guiding questions
Intersecting Categories:	Has the combination of different social factors, such as age, gender, race/ethnicity, class, and migration, been addressed in identifying the causes of the problem?
Multilevel Analysis:	Have the various factors at the individual, interpersonal, organizational, and governance levels been addressed in the process of problem identification? Have the intersections of social factors across micro, meso, and macro level been considered?
Power:	Have stakeholders such as affected populations participated in problem identification? Have the structures of power such as policies and laws been addressed to be responsible for the framing of the health problem? Has the intervention/program been framed within the current cultural, political, economic, and societal context? And has it reflected the needs of affected populations?
Reflexivity:	Do the planning committee/research team look critically at their values, experiences, beliefs, and assumptions, about the health problem? Do the researchers/health planners have reflexive practice?
Time and Space:	Has the process of problem framing over time (historically) or across different places (geographically) and changes of privileges and disadvantages, including intersecting identities and the processes that determine their value over time and place, been considered? Is the intervention/program flexible in terms of time and place conditions?
Diverse Knowledge:	Has the perspective of people who are typically marginalized been used in the process of problem identification? Has the knowledge been generated from several resources including qualitative or quantitative research; empirical or interpretive data; and Indigenous knowledge? Has the target group’s knowledge been used in the process of health program design and implementation?
Social justice and Equity:	Do current interventions/programs focus on the health promotion of vulnerable groups? Has intervention/program been designed and implemented to reduce inequalities?

### Quality assurance

We have added as an Additional File (Additional file 1) the Consolidated Criteria for Reporting Qualitative Studies (COREQ) Checklist [35], which includes relevant details regarding the team and the research process. During the data collection and analysis, we triangulated [36] across methods and participants. Including this strategy enhanced the intersectionality efforts, as the perspectives are complementary and allow us to gain insight into a broader picture of the situation. We applied peer debriefing among the researchers and with collaborators from MoH throughout the data collection and data analysis process [32, 36].

## Results

Observations, interviews, focus groups, workshops, and literature reviews have yielded a complex picture of the situation of EMTCT plus diseases in Comapa. The relationship between the different stakeholders, as well as with midwives, reveals the functioning of the healthcare system. Sociopolitical and historical factors are crucial in understanding the current social role of midwives in their context. We argue that understanding the interactions between the different knowledge systems (and the meanings of health, illness, and nature) determines the success of public health policies on the treatment, diagnosis, and administration of EMTCT Plus diseases and mother–child health in general.

### Health care system

#### Stakeholders at multiple levels shaping the health care system

**Fig. 4:**
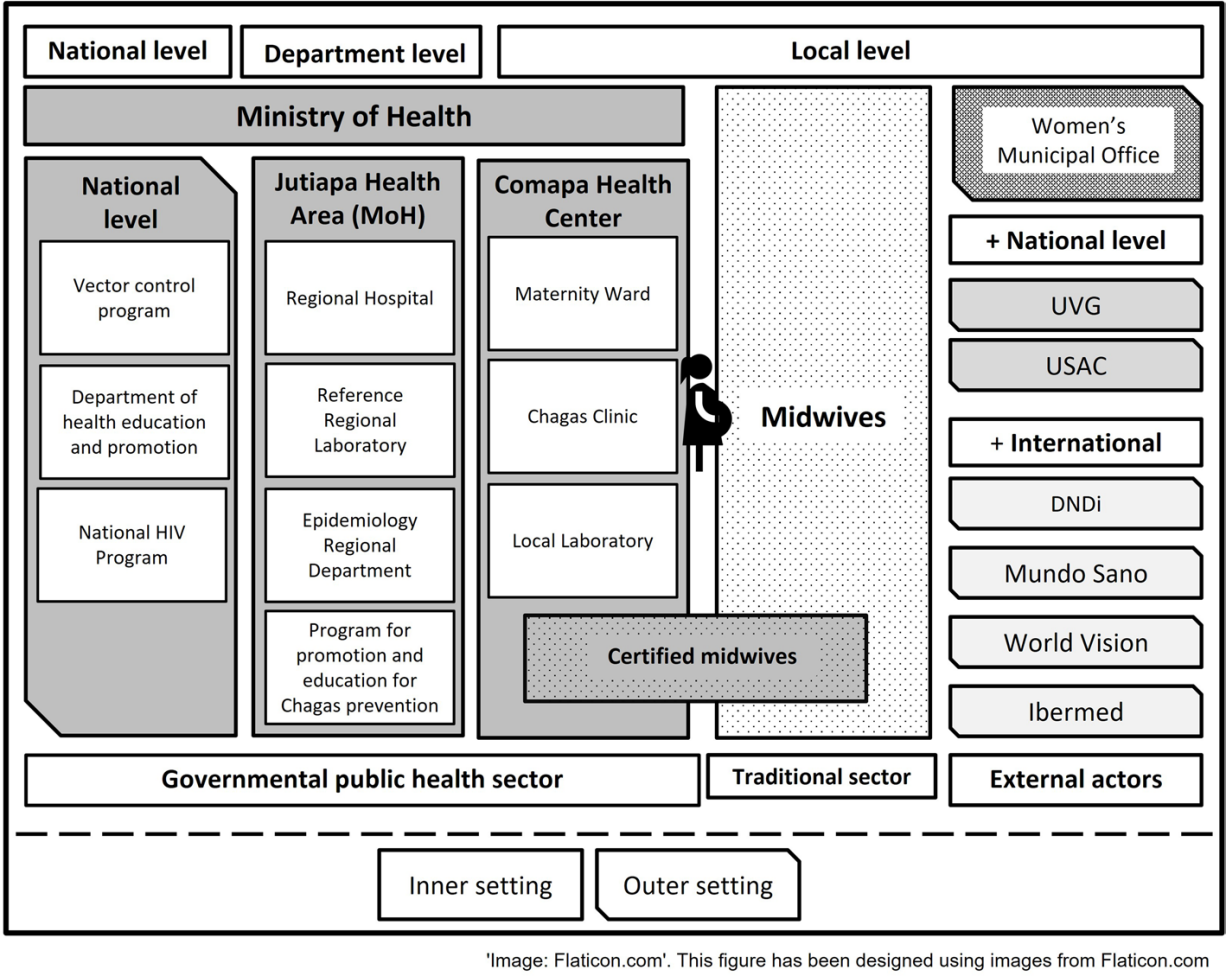
Stakeholders Map. This figure shows the institutions and actors involved in perinatal care at the local, department, and national levels. The figure shows the dichotomy between the public health sector and the traditional sector (midwives), as well as external factors (‘outer setting’) identified at the local level, on which our research focused. *USAC is Universidad de San Carlos de Guatemala, UVG is Universidad del Valle de Guatemala and DNDi is Drugs for Neglected Diseases initiative. Although not exhaustive, this map shows the stakeholders identified at the time of the study and potential collaborators

We developed a stakeholder map (Fig. 4), crucial for subsequent procedures, to guarantee transversal participation in the implementation of health policies (prevention, diagnosis, treatment) related to EMTCT Plus.

The major stakeholder determining the content and direction of health-related initiatives in Guatemala is the MoH. For the case of EMTCT Plus diseases, the MoH has developed programs for prevention, diagnosis, and treatment that cover vector control, epidemiological initiatives, and health education and promotion at the national, regional, and local levels. These programs are executed at the departmental and local levels by the regional and local health care centers (i.e., regional hospitals, reference laboratories, epidemiology departments, and local Chagas management programs). At the very local community level, the Chagas Clinic, maternity wards, midwives, and local laboratories are responsible for enacting national and regional directives.

Furthermore, acting at local, national, and international levels, it is possible to find nongovernmental organizations that take on public health roles following a personal or global agenda (such as DNDi, World Vision, Mundo Sano, Ibermed, and others). The actors identified in Fig. 4 contribute in different ways to the prevention, diagnosis, and treatment efforts of EMTCT Plus diseases. The interaction of the different actors influences the configuration of what is understood as the public health system, an ambiguous concept not easily defined but essential in the creation and implementation of health policies.

### Midwives as key stakeholders in the implementation of EMTCT programs

In 2015, CARE International registered an average of 23,000 midwives in Guatemala, 16,000 of whom had received formal training from the official health system [37]. These midwives attended two-thirds of all births in Guatemala, 80% of which were in rural Mayan areas [12]. Much of the work focusing on Guatemalan midwives has been conducted in the western highland areas among populations who identify themselves as Mayan. Given that the population in southeastern Guatemala has a less well-defined self-identification with indigenous groups, studies on alternative health systems tend to leave these populations out, except for the work of Bossen (1984) and Cominsky (2016) [12, 38].

The midwives who participated in the interviews and focus groups were approximately 60 years old, and many had been assisting an average of four women per year for more than 10 years. However, there is no homogeneous profile of midwives in Comapa. They all have different levels of education (not all of them learned to read or write), and they have different roles within the community depending on their participation in other groups such as the church, an NGO, or the local government. Some of them also practice other forms of alternative healing, extending their knowledge and their role within the community.

Becoming a midwife in the Comapa region does not follow the same trajectory as in the Mayan highlands on the western side of the country, where women receive their calling often in dreams [12]. Midwives in our study area have received training from their grandmothers or even from the local health care center, and they do not necessarily gain their livelihood solely from their midwifery work. The women they assist pay a symbolic wage when possible. They are sometimes selected by the local leaders at the community development council (COCODE—*Concejos Comunitarios de Desarrollo*) for their leadership capabilities [39].

Not all midwives in Comapa attend the training provided by the local health care center. Consequently, not all of them can register newborns in the RENAP (National Registry of Persons). Similarly, not all of them have technical training to identify risks and recommendations for pregnant women. Although midwives receive information about EMTCT Plus during local health care center training, they are not trained to screen pregnant women or newborns in a community setting. However, they often provide important information to their patients and their families, mainly about sexual and reproductive health. The Comapa local health care center does not share the results of the tests performed on pregnant women and newborns with midwives, and each woman decides whether to share the information personally with their midwife. Midwives who have received training at the local health care center and accompany pregnant women throughout pregnancy and postpartum provide emotional support and share traditional and some biomedical knowledge. In the case of Chagas in Comapa, there are multiple organizations and institutions involved, from vector control to diagnosis and treatment, as shown in the stakeholder map (Fig. 4). Not all areas of Guatemala display such a complex network of Chagas-related actors.

### Diverse forms of knowledge

Given that in 2018, 25% of the total population above 4 years old had no level of formal education, there are high illiteracy rates in Comapa [27], and any educational and communication material must be visually clear and reflect local gender and ethnic views. Additionally, midwives in Comapa manage a combination of knowledge systems around health and wellbeing. While they do not identify as Mayan, the influence of Xinka beliefs has permeated through their understandings of health, social relations, and notions of well-being that extend beyond the biomedical individualist conceptualization of health and healing [40]. The Mayan belief that a body must be in balance within itself, as well as with the community and environment [41], and the importance of balancing cold-hot states within the body were evident concepts emerging through interviews with midwives in Comapa.

Midwives represented their conceptions of health and healing through visual expressions. These were classified into the following categories: (1) nature, (2) social role, and (3) the local health care center, according to the themes that emerged in the interviews. The drawings were also interpreted in relation to interviews and informal conversations with midwives. These emic views of health and well-being highlight characteristics that must be considered in the elaboration of educational and communicational material.

### Nature

The prominent presence of nature in the drawings – linked to flora and fauna – was explained by the midwives as a symbol of life. Furthermore, plants are also used to cure ailments, as midwives receive knowledge of the medicinal properties of plants from their grandmothers. Animals were also drawn as they constitute an important presence in daily life: donkeys serve as transport in remote areas or are used to till the fields, chickens provide eggs, pigs provide meat, cats keep the houses free of mice that would otherwise eat the stock of maize (central for subsistence and rich in symbolic value).
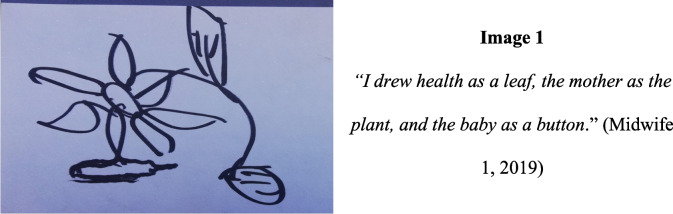


### Social role

The category of the social world was constructed from midwives’ accounts of the impact of their activities on the lives of others in their community, which granted them value as healers within a social context.
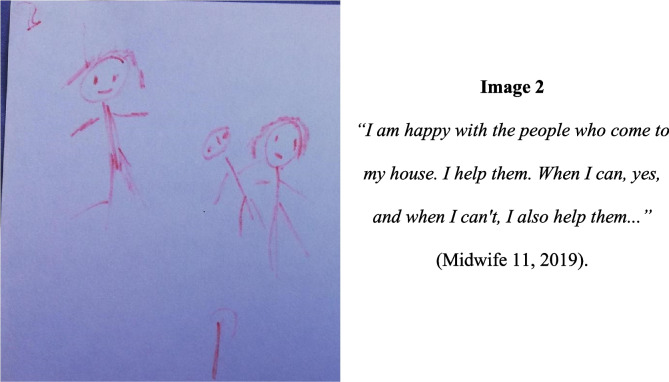


This comment must be understood in the context of the importance of support and sympathy in the process of healing. Healing in Maya practices has a large relational component [41], which depends on the immediate family, as well as the larger community and the ancestors, becoming involved in the healing processes. Therefore, even when midwives may not have the tools or training to directly address the immediate health problem they get consulted about, they act as enablers and support in the process of redressing the imbalance that illness causes.
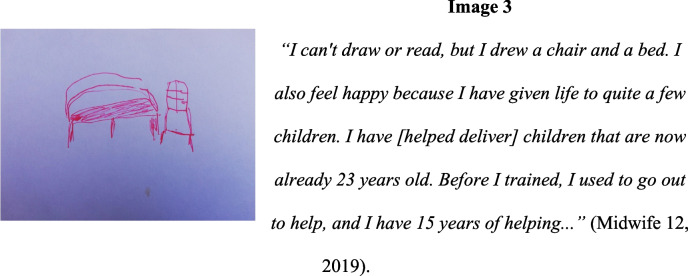


### The local health care center

While midwives sometimes get basic first aid materials from the local health care center, their main ‘tools’ are their hands, through which they feel the pregnant women’s bellies and the position of the babies before birth.
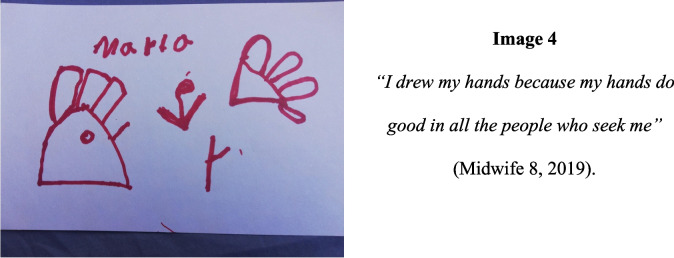


The following comment, which was not an isolated one, points to the structural hierarchies reproduced in the training sessions received by the midwives, where the single most important message is the referral of women to the local health care center.
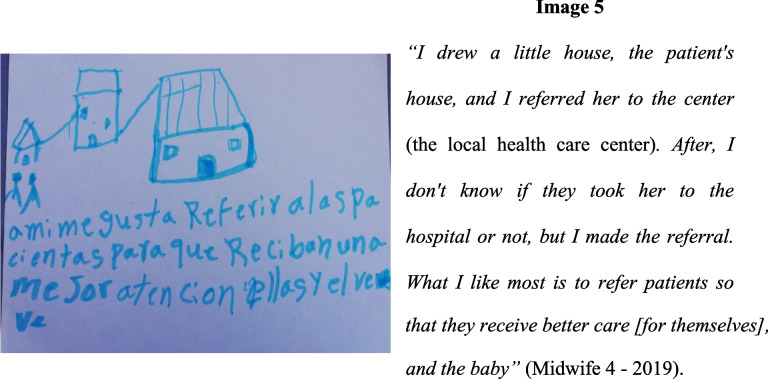


### Diagnosis, prevention, and treatment protocols

**Fig. 5:**
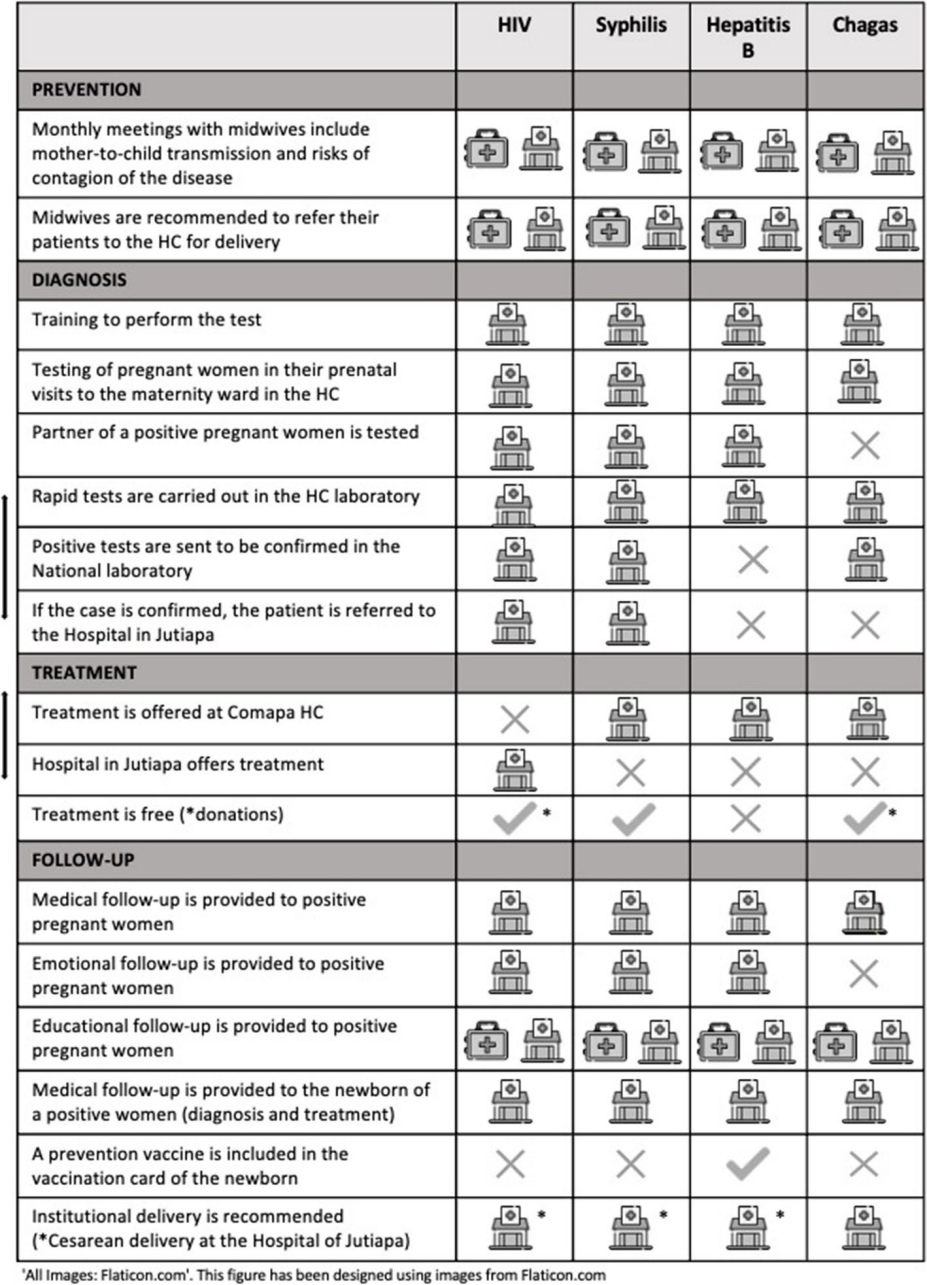
Comparison of prevention, diagnosis, treatment, and follow-up routes for the four diseases included in EMTCT Plus

Despite being part of an integrated EMTCT Plus official strategy, these four diseases are not yet addressed through a unified protocol, even though in practice, there are many similarities in the way they are approached (Fig. 5). In the prevention stage, which refers mainly to the education and attendance of pregnant women at the local health care center, medical staff (doctors and nurses) and midwives jointly work to inform the patients and refer them for institutional delivery. The local health care center is mostly in charge of the diagnosis stage, in which rapid tests are performed to screen the four diseases in Comapa’s laboratory, and the positive samples are sent to the laboratory in Jutiapa and later to the national laboratory for confirmation.

Regarding the treatment of positive patients, syphilis, hepatitis B, and Chagas are treated at the local level at the local health care center; however, HIV-positive patients are referred to Jutiapa Hospital at the department level. Treatment for HIV, syphilis, and Chagas is free of charge, and the availability for HIV and Chagas treatments is provided by the MoH and must be processed by the public health system. While syphilis should be treated during pregnancy to prevent malformations of the fetus (and both mother and father should be treated to prevent reinfection), Chagas treatment cannot be given until after birth and the breastfeeding period. The follow-up of patients is provided jointly by midwives and local health care center personnel. Institutional delivery is recommended for women seropositive for any of the four diseases, with HIV, syphilis, and hepatitis B requiring cesarean delivery in the departmental hospital, as they are not yet equipped to perform them at the local level. Institutional delivery also allows for medical follow-up of newborns, which includes a vaccine for hepatitis B.

Figure 5 shows both the activities performed by the Comapa local health care center and the midwives. The table is divided according to the major perinatal activities, which also represent some of the key stages of ETMTC Plus goals. The first aid kit icon represents the role of midwives, while the clinic icon represents duties implemented by the Ministry of Health at its different levels.

The results shared by the laboratory in Jutiapa (Table 2) point to a difference among screening strategies for pregnant women in Comapa concerning all EMTCT Plus diseases. While the screening and monitoring of these diseases are officially under the responsibility of the local health centers, midwives are involved mainly in the prevention and follow-up stages. Sometimes, midwives accompany patients and their families in the predelivery, delivery, and postdelivery stages. Although midwives have been trained at the local health care center to encourage their patients to have assisted institutional births, many times patients do not reach the official facilities due to emergencies, lack of time, accessibility problems, or personal decisions. The records of births and follow-ups of pregnant women kept at the Regional Laboratory in Jutiapa Hospital concerning tests and births registered in Comapa showed that, even though women go to the local health care center for check-ups, many decide to continue attending their pre- and postpartum care with the midwives. These interactions are not mutually exclusive.

**Table 2:**
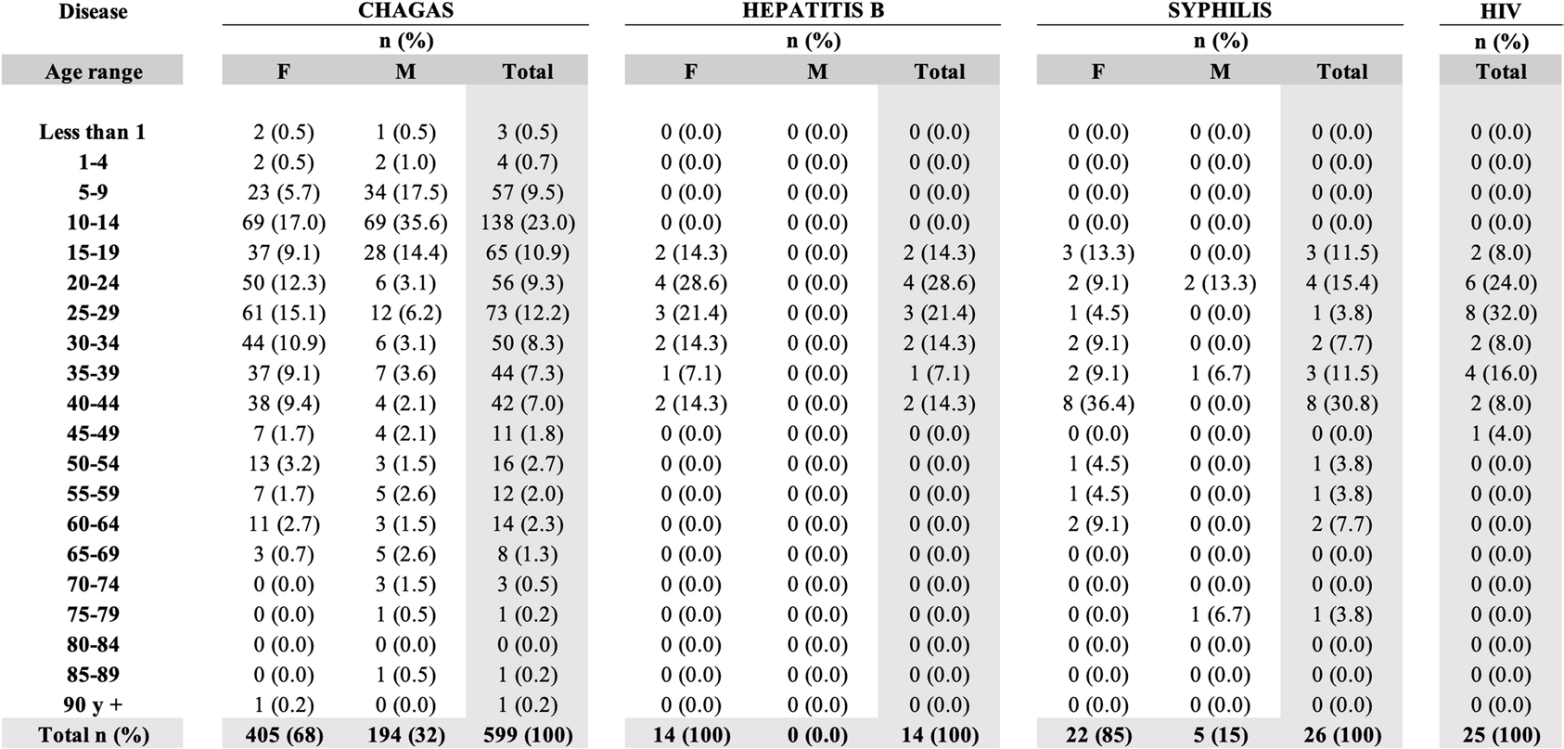
Total of samples analyzed in the Jutiapa laboratory from Comapa local health care center between 2012 and 2019 by disease and sex

### Epidemiological data: HIV, syphilis, hepatitis B, and chagas disease

We present the data shared by the laboratory at Jutiapa between 2012 and 2019, focusing specifically on the samples collected in Comapa, as it is the municipality where our work took place. The disease with the most samples taken for confirmation was Chagas disease, followed by syphilis and HIV, with only 14 samples taken for hepatitis B.

When analyzing the data by sex, most samples for the four diseases were obtained from women, representing over two-thirds of the total samples taken for Chagas and syphilis. All samples from hepatitis B were from women. Data from HIV patients were not specified by gender, and data were not shared. Having mostly female records for all four diseases suggests that men do not attend testing as often as women or that men are not targeted by the healthcare system in screening protocols. Promotion from the MoH together with the Universidad del Valle for mother and child Chagas Disease screening has been persistent since 2015 [14] which could explain the difference in the quantity of screened men and women in comparison to the other three diseases. However, the inclusion of men would be important, considering that some of the EMTCT Plus diseases are sexually transmitted. Particularly in the case of syphilis, the reinfection of women during pregnancy can result in birth defects for the baby.

Most of the test results for the four diseases were performed on people between the ages of 15 and 50 (women’s reproductive age). In addition, the high number of men and women between 10 and 14 years of age screened for Chagas may correspond to screening activities conducted in schools. Regarding data from newborns and children, there were tests reported only for Chagas disease.

## Discussion

One of the biggest challenges in the implementation of health programs is the complexity of the concepts of health and disease in culturally diverse contexts [42]. Therefore, the success or failure of an intervention is largely dependent on its cultural appropriateness, that is, on its capacity to consider the particularities of the target group, its acceptance within the community, and its sustainability [20, 43].

The burden of social determinants of health on the diagnosis, treatment, and prevention of EMTCT Plus diseases demands a focus on social factors in the design and implementation of this health strategy. Historical, economic, political, and institutional characteristics influence a person’s degree of vulnerability to disease. This implies “having sufficient knowledge of the mechanisms influencing health inequities and adopting a conceptual framework that not only clarifies the relationship between social determinants and health inequities but also helps to identify entry points for intervention” [44].

Additional complexity is provided by the multiplicity of cultural patterns found within the region, requiring not only attention to general structural determinants of health, such as poverty, illiteracy, or labor status but also to more nuanced cultural features, such as land tenure conflict, alternative notions of health and illness, and different kinship patterns [45]. The targeted and tailored approach to health promotion programs described by Kreuter et al. (2003) proposes interventions tailored for “a defined population subgroup that takes into account characteristics shared by the subgroup’s members” and makes use of five strategies to carry out a program in a culturally tailored manner [17, 25, 46]. These strategies (mentioned in Fig. 2) help address the intersections, complexity, and heterogeneity of people in a community based on individual factors, experiences, and their relationship with the broader system [20]. They also generate a more intersectional approach by demanding knowledge interactions between different sectors and disciplines and a consultative process with all stakeholders [25].

Recalling that intersectionality can serve as a method – described above – as well as an analytical tool [21, 46], we now focus on this second aspect of the concept by identifying the categories highlighted by Ghasemi. et al. (2021) emerging from our project design and implementation: intersecting categories, multilevel analysis, power, reflexivity, time and space, diverse knowledge, and social justice and equity [18, 20].

The cultural context and historical framing of the current project have been the starting point of the research agenda of the interdisciplinary team. The production data collection process captured the historical background and cultural characteristics of the local inhabitants of Comapa, produced through the consultation of archival material and field research, and shed light on social, economic, and political categories shaping the experiences of health in the region. Since social stratifying categories (ethnicity, gender, race, social status) are closely linked to other determinants of health (economic, environmental), an intersectional analysis reveals how different social stratifies interact with one another to condition access, prevention, control, and management of health [19, 20, 25].

A focus on midwives and vertically transmitted diseases meant that our research participants were primarily women. Working in collaboration with local midwives is imperative to promote ethnic and gender equity through the development of culturally appropriate educational and communication material. However, given the patriarchal structures of the rural communities in Comapa, we found ourselves often having to talk to men and needing to be accompanied in the field by a male fieldwork technical staff, as we were mostly female researchers. Thus, we unpack gender categories from the inception of the research project [20, 45].

Male involvement in health-related processes (specifically EMTCT Plus disease processes) is a complex issue. Midwives interviewed mentioned prohibitions imposed by husbands on their wives that relate to pregnancy management: birth control, capacity to attend pregnancy controls at the local health center, inability to attend the hospital without male patronage, and lack of permission of midwives to see the pregnant women without the husband’s consent or presence. For women, refusing to comply with these types of prohibitions means placing themselves in vulnerable scenarios that could also have repercussions for the midwives. One response to this situation is for the women to visit the local health care center without the knowledge of their partners, with the support of the midwives and nurses, who keep the women’s ID cards so that their husbands/partners cannot find them. A multilevel analysis (individual, interpersonal, organizational, and national) reveals that access to health care is dependent not only on infrastructure but also on social determinants and cultural patterns [20, 25].

Success in preventing and controlling EMTCT Plus diseases requires socioculturally appropriate and gender-sensitive strategies that engage local stakeholders and address ethnic, gender, and economic equity. It is not always easy to identify which essential players need to be involved in the process for it to be socioculturally appropriate and gender sensitive. The participation of multiple stakeholders, from midwives to the national level of health with whom we maintain close dialog throughout the design and implementation of this project, has aimed to produce consistent, respectful, and culturally appropriate content that should have acceptance at the community level, as well as be consistent with the national policy guidelines around EMTCT Plus [18, 20]. Through the application of **evidential strategies**, we found that most actions around diagnosis and treatment were already in place for the four diseases; therefore, our proposal focuses on the development of educational and communication material strategies that integrate and intersect HIV, syphilis, hepatitis B, and Chagas disease from a congenital perspective [17]. Furthermore, the challenges of consistently implementing a homogeneous testing strategy may result in an uneven application of healthcare practices, as well as the different levels of equipment of the various health care centers. Uneven population densities, access roads, and multiple social and geographic conditions affecting each locality determine the number of people who make it to the local health care centers as well as their demographic characteristics [45]. This is important to note when developing screening and treatment programs so that they consider the particularities of each locality. Therefore, proposing the development of educational and communication material that integrates testing and perinatal care algorithms can strengthen the current services offered to pregnant women. We have taken Comapa as our case to analyze its local characteristics and how they demand special attention when designing health interventions.

The use of **constituent-involving strategies** that consider processes of community engagement was applied throughout the entire development of the project. Additionally, our team had worked during previous years applying community engagement strategies focused specifically on congenital Chagas disease [14, 17]. This participatory model served as a base for the EMTCT efforts, as some relationships and rapport were already built with personnel from the MoH and other government and nongovernment institutions related to maternal-infant health issues, including midwives at the core of the efforts [25]. While the participation of different stakeholders through a consultative process has been a fundamental conceptual and methodological tenet of this research, we have had to navigate the multiple power struggles and hierarchies present between different knowledge systems and other social and cultural tensions [10, 34]. It is not easy to transform social and cultural patterns, but we agree with Hankivsky et al. (2010) that through intersectionality and gender-sensitive research, it is possible to overturn structural inequalities by developing methods that elucidate power and have the potential for social change [47].

The stakeholders' map (Fig. 4) served to understand the power dynamics that facilitate or hinder the roles of each actor. Although there is a national law regulating midwives’ activities, at the local level, midwives often encounter multiple barriers. For example, from the MoH perspective, only the midwives who attend a certain number of workshops at the local health care center are officially certified. Therefore, another layer of unequal power dynamics emerges between “certified” midwives and “empirical” midwives [25]. Having worked only with the “certified” midwives, we have had to exclude the perspectives of “empirical” midwives as well as the women they attend.

The constant consultation with the community, organized through the personnel of the local health care center, has the intention of facilitating and encouraging communication channels between the different health agents. Therefore, while centralizing events (focus groups, consultations, and work with accredited midwives) may imply the reproduction of preexisting hierarchies of power, it also aims to overturn these hierarchies from within through the very intersectional methodology [10, 14, 25, 34].

The work with midwives was also part of our **sociocultural strategies**, which aimed to identify and consider the particularities of the target group. Although there has been an increase in pregnant women attending the local health care center for their prenatal check-ups and delivery, there are still many women who continue to seek midwives for pregnancy-related care, whether from their community or a neighboring one. A preference for midwives may result from the value of their alternative knowledge and more personalized service, but it can also be related to the lack of access to institutional healthcare services [16, 45]. For example, in some communities, there are no adequate roads to easily reach the local health care center, and finding transportation can have an economic cost for the patients and their families. Midwives also incur a cost for the families, as opposed to the local health care center, which is free of charge. This implies that economic valuations play a role in people’s decisions when seeking health assistance.

Midwives fill gaps left unaddressed by public health institutions, which do not always reach the whole population. However, they cannot exist independently from it. For example, midwives are not trained to take blood samples, which is part of the EMTCT Plus goals. Therefore, the strategy developed understands the complementarity of the two systems, in this case, enhancing midwives' referral of women to the local health care center for their perinatal care and delivery but empowering them to be the first port of call for questions about EMTCT Plus diseases [14].

A strategy to ensure this has been the development of educational and communication material to be used by the different stakeholders (public health institutions, NGOs, and midwives) targeting different audiences: healthcare personnel in their education of midwives and midwives in their transference of the message to pregnant women [14, 17, 20]. Considering that midwives could have a key role in the implementation, the material should include their sociocultural perspectives and their knowledge and should be adapted to their literacy level and preferred language. Using **linguistic strategies** and **peripheral strategies** helped us tackle these issues [17]. The development and validation of the material is part of the following stage of the project, currently underway.

## Conclusion

To conclude, we outline key contributions resulting from a participatory research project that takes gender intersectionality as a methodological and analytical tool as central to improving access to maternal and child health screening services.

First, the integration of the theoretical perspectives of intersectionality and social determinants of health in an illustrative figure (Fig. 1) has aimed to clarify the practical implementation of these theories in programs of cultural tailoring. Second, the development of a stakeholder map (Fig. 4) of relevant actors is transferable for the implementation of other community development initiatives, with its context-specific adjustments. Identifying important local, national, and regional actors that should be coordinated in implementing health programs is a complex task that can only be achieved through fieldwork, long-term engagements within the field, and building respectful relationships with multiple partners, ideally from diverse disciplines. This process unpacks the structures of the healthcare environment but needs to consider the people who fill the positions, as these changes are susceptible of political shifts which may influence the predominance of certain structures over others. Third, the identification of the diagnosis, prevention, and treatment routes for the four EMTCT Plus diseases (Fig. 5) was a useful and potentially transformative contribution, as it enables the improvement of future health practices supporting women’s and children’s health. The development of a unified and integrated protocol for diagnosing and treating these vertically transmitted diseases is the focus of ongoing projects at the partner NGO, and empirical research such as this, which produced relevant data for the development of public policy, is a valuable contribution.

Finally, although not extensively, this project has aimed to demonstrate a possible way of implementing culturally tailored health programs designed through a participative methodology, sensitive to gender issues, and intended upon the creation of concrete deliverables for the use and benefit of the target community (educational and communication material for midwives to use with the women they provide care to and for the local health care center to use in the training of midwives). A key purpose of these materials will also be the shifting of power relations within the conventional biomedical system, where midwives (and their alternative knowledge) are often subsumed under the hierarchy of biomedical science. In the upcoming phase of development of the materials and their pilot use within the community through participatory methods, we hope to engage the different actors in conversations that generate space for dialogue in a more equal standing. Whether this can be achieved remains to be seen. The importance of such an objective is that the methodology itself is a key element in the pursuit of the goals of the project and has a transformative intention.

### Supplementary Information

**Additional file 1:** **Supplemental File 1. **Applying the Consolidated Criteria for Reporting Qualitative Studies (COREQ): 32-item Checklist to our study.

**Additional file 2:** Spanish translation of the article.

## Data Availability

The data we used and/or analyzed for the present study are available upon reasonable request through the corresponding author to respect the IRB confidentiality agreement that does not allow individual disclosure of data. A Spanish translation of this article is available (see Additional file 2).

## Abbreviations

EMTCT Plus: Elimination of mother-to-child transmission of HIV, Syphilis, Hepatitis B, and Chagas
PAHO: Pan American health organization
HIV: Human immunodeficiency virus
STD: Sexually transmitted diseases
MoH: Ministry of Health
SDH: Social determinants of health
COREQ: Consolidated criteria for reporting qualitative studies
USAC: Universidad de San Carlos de Guatemala
UVG: Universidad del Valle de Guatemala
DNDi: Drugs for neglected diseases initiative
COCODE: Concejos Comunitarios de Desarrollo
RENAP: Registro Nacional de Personas
