# Correction: Incorporating an intersectional gender approach to improve access to maternal and child health screening services

**DOI:** 10.1186/s12939-024-02161-z

**Published:** 2024-05-16

**Authors:** Margarita Rivera Arrivillaga, Marina Gold, Elizabeth Pellecer Rivera, Jose Guillermo Juárez

**Affiliations:** 1 Centro de Estudios en Salud, Universidad del Valle de Guatemala, 18 Av. 11-95 Zona 15 VH III, Guatemala City, Guatemala; 2 Fundación Mundo Sano, Recaredo, 3. Puerta Garaje, 28002 Madrid, Spain; 3 Environmental Sciences, University of Maine, 04469-5755 Orono, ME USA

International Journal for Equity in Health (2024) 23:32

10.1186/s12939-024-02109-3

After publication of this article, the authors reported that in the Funding information section the following was missing: ‘Earlier stages of the work presented here were financially supported by the Centers for Disease Control and Prevention (CDC) and by the UNICEF/UNDP/World Bank/WHO Special Programme for Research and Training in Tropical Diseases (TDR) ’.

The original article has been updated by the authors.

