# Supplementary material for: Incorporating an intersectional gender approach to improve access to maternal and child health screening services
Source: Int J Equity Health. 2024 Feb 20;23:32. doi: 10.1186/s12939-024-02109-3 (PMC10880357; doi:10.1186/s12939-024-02109-3)
Supplement: Supplementary file 1 — Additional file 1: Supplemental File 1. Applying the Consolidated Criteria for Reporting Qualitative Studies (COREQ): 32-item Checklist to our study. [file 12939_2024_2109_MOESM1_ESM.docx]

**Supplemental File 1. Applying the Consolidated Criteria for Reporting Qualitative Studies (COREQ): 32-item Checklist to our study.**

| **No** | **Item** | **Guide Questions** | **Description and/or Reference in the Article** |
| --- | --- | --- | --- |
| **Domain 1: Research team and reflexivity** | | | |
|  | ***Personal Characteristics*** | | |
| 1 | Interviewer/ facilitator | Which author/s conducted the interview or focus group? | Researcher 1 (R1) conducted interviews.  Researcher 2 (R2) conducted interviews, workshops, and focus groups.  Researcher 3 (R3) supported the design of the data collection instruments and the analysis.  Researcher 4 (R4) supported the workshops, logistics, and focus group. |
| 2 | Credentials | What were the researcher's credentials? E.g., PhD, MD | R1, R2 and R3 are anthropologists, R1 had a PhD, R2 a BA degree, and R3 a MA degree. R4 is a field technician. |
| 3 | Occupation | What was their occupation at the time of the study? | R1, R2, and R3 were social research assistants for the project and R4 was a field technician for the project. |
| 4 | Gender | Was the researcher male or female? | Both R1, R2, and R3 are female. R4 is male. |
| 5 | Experience and training | What experience or training did the researcher have? | R1, R2, and R3 are social scientists, they had experience designing, conducting, and analyzing qualitative research. R4 had over a decade of experience as a field technician and has been working in the region since 2010. |
|  | ***Relationship with participants*** | | |
| 6 | Relationship established | Was a relationship established prior to study commencement? | The relationship between our team and the stakeholders in Comapa began in 2015, with a project focused on congenital Chagas. However, some of the staff from our organization changed, so a direct rapport with them began in 2019. |
| 7 | Participant knowledge of the interviewer | What did the participants know about the researcher? e.g., personal goals, reasons for doing the research | The participants were informed about the research objectives and all the information included in a consent, prior to their involvement with the project. All activities provided a space for participants to ask the questions they might have had.  Given that the visits to the area and the activities were ongoing, the exchange of personal information also happened (i.e., about the family, personal concerns, personal activities, etc.). |
| 8 | Interviewer characteristics | What characteristics were reported about the interviewer/facilitator? e.g., Bias, assumptions, reasons, and interests in the research topic | The first language of all the researchers is Spanish, and all data was collected in Spanish.  R2 and R4 have previously worked in the region. |
| **Domain 2: Study design** | | | |
|  | ***Theoretical framework*** | | |
| 9 | Methodological orientation and Theory | What methodological orientation was stated to underpin the study? e.g., grounded theory, discourse analysis, ethnography, phenomenology, content analysis | Overall, we used an intersectional gender approach for data collection and data analysis, applying the Cultural Appropriateness Framework (Kreuter et al., 2003) and the categories for intersectional analysis proposed by Ghasemi et al. (2021). |
|  | ***Participant selection*** | | |
| 10 | Sampling | How were participants selected? e.g., purposive, convenience, consecutive, snowball | MoH staff and external collaborators:  We used a purposive sampling, targeting key stakeholders working in maternal and infant health.  Six semi-structured interviews were conducted with representatives of the MoH staff at department and municipal level.  Three semi-structured interviews were conducted with external collaborators working on related issues.  Midwives:  We also used purposive sampling with this group, based on the criteria of their attendance to the MoH organized activities.  We conducted one participatory workshop with 19 midwives; one focus group with 13 midwives; and four semi-structured interviews. We visited the houses of 12 midwives for participant observation.  Meetings with staff at the central Ministry of Health unit and other key stakeholders. |
| 11 | Method of approach | How were participants approached? e.g., face-to-face, telephone, mail, email |  |
| 12 | Sample size | How many participants were in the study? |  |
| 13 | Non-participation | How many people refused to participate or dropped out? Reasons? | No participants refused to participate or dropped out. |
|  | ***Setting*** | | |
| 14 | Setting of data collection | Where was the data collected? e.g., home, clinic, workplace | We conducted the activities in the participants’ house, office, and in the local health care center. The participants decided what location was convenient for them. |
| 15 | Presence of non-participants | Was anyone else present besides the participants and researchers? | In the health care center, sometimes there would be other nurses or personnel present. In the houses, sometimes there would be other family member present. |
| 16 | Description of sample | What are the important characteristics of the sample? e.g., demographic data, date | The MoH staff at the municipal level is mostly female. Nurses are female, but staff at the vector control unit for Chaga are mostly male.  The midwife’s group is all female, above 40 years old. Their literacy level varies. |
|  | ***Data collection*** | | |
| 17 | Interview guide | Were questions, prompts, guides provided by the authors? Was it pilot tested? | All the instruments were designed by R1, R2, and R3.  There were 3 semi-structured interview instruments, corresponding to the target group, and 2 instruments corresponding to the focus group and workshop. |
| 18 | Repeat interviews | Were repeat interviews carried out? If yes, how many? | We did not repeat any interview. |
| 19 | Audio/visual recording | Did the research use audio or visual recording to collect the data? | The interviews and group activities were audio recorded, after obtaining participants consent. |
| 20 | Field notes | Were field notes made during and/or after the interview or focus group? | The researchers took field notes during their data collection. Additionally, we compiled this information into reports throughout the data collection period. |
| 21 | Duration | What was the duration of the interviews or focus group? | Interviews lasted between 20 and 40 minutes. The workshop and focus group lasted one morning. The duration of the house visits was 20-30 minutes. |
| 22 | Data saturation | Was data saturation discussed? | Data saturation was not discussed by the team.  We reached data saturation. |
| 23 | Transcripts returned | Were transcripts returned to participants for comment and/or correction? | Transcripts were not returned nor discussed with the participants. |
| **Domain 3: analysis and findings** | | | |
|  | ***Data analysis*** | | |
| 24 | Number of data coders | How many data coders coded the data? | The data was coded by three researchers, R1, R2, and R3. |
| 25 | Description of the coding tree | Did authors provide a description of the coding tree? | Not applicable |
| 26 | Derivation of themes | Were themes identified in advance or derived from the data? | Themes were derived from the data, particularly interviews and focus group. |
| 27 | Software | What software, if applicable, was used to manage the data? | No software was used to process data. Data was stored in Google drive, password protected, and shared only among members of the project. |
| 28 | Participant checking | Did participants provide feedback on the findings? | The participants did not provide feedback on the findings. However, the participants are still involved in the project in the development of the educational material, which is being developed in consultation with participants. |
|  | ***Reporting*** | | |
| 29 | Quotations presented | Were participant quotations presented to illustrate the themes / findings? Was each quotation identified? *e.g., participant number* | Yes, some quotations and drawings generated by the participants are included in the Results section. The quotations are identified with a code, for confidentiality. |
| 30 | Data findings consistent | Was there consistency between the data presented and the findings? | Yes, we believe the data presented are consistent with the findings. We are using the information contained in this manuscript to develop educational material on the topic. |
| 31 | Clarity of major themes | Were major themes clearly presented in the findings? | Yes, the major themes were presented in the Results section, along with the minor themes identified. |
| 32 | Clarity of minor themes | Is there a description of diverse cases or discussion of minor themes? |  |
