# Supplementary material for: Incorporating an intersectional gender approach to improve access to maternal and child health screening services
Source: Int J Equity Health. 2024 Feb 20;23:32. doi: 10.1186/s12939-024-02109-3 (PMC10880357; doi:10.1186/s12939-024-02109-3)
Supplement: Supplementary file 2 — Additional file 2: Spanish translation of the article. [file 12939_2024_2109_MOESM2_ESM.pdf]

Incorporación de un enfoque interseccional de género para mejorar el acceso a los servicios de tamizaje en salud materno infantil

Margarita Rivera Arrivillaga[1*], Marina Gold[2*], Elizabeth Pellecer Rivera[1], José Guillermo Juárez[1].

1 Centro de Estudios en Salud Universidad del Valle de Guatemala, 18 Av. 11-95 Zona 15 VH III, Guatemala City, Guatemala
2 Fundación Mundo Sano, Recaredo, 3. Puerta garaje 28002 Madrid, España.

Resumen

Antecedentes

La Organización Panamericana de la Salud (OPS) ha impulsado iniciativas que buscan la eliminación de las enfermedades de transmisión materno infantil en las Américas desde hace más de dos décadas. Si bien Guatemala ha asumido el compromiso de mejorar el acceso y la cobertura de los servicios reproductivos y perinatales, las metas aún no se han alcanzado. Muchas veces, la implementación de estos esfuerzos se ve obstaculizada por complejidades arraigadas en intersecciones sociales, culturales y ambientales. Este estudio muestra una estrategia novedosa que incorpora la interseccionalidad como un componente metodológico y analítico. Esto contribuye a evidenciar la manera en que la interseccionalidad y la participación comunitaria pueden ser aplicadas para fortalecer los esfuerzos de salud pública en torno a la implementación de la iniciativa ETMI Plus (Eliminación de la transmisión materno infantil del VIH, sífilis, hepatitis B y enfermedad de Chagas), en el municipio de Comapa, en Guatemala, el cual es mayoritariamente rural.

Métodos

Implementamos una metodología participativa, integrando marcos teóricos y metodológicos para comprender la prestación de servicios de salud, tanto por parte de comadronas como de la institución de salud pública, desde una perspectiva interseccional para la prevención, diagnóstico, tratamiento y seguimiento de VIH, sífilis, hepatitis B y Chagas. Los datos fueron recolectados a través de entrevistas, grupos focales, talleres y tras la revisión de bases de datos de laboratorio, y nos guiamos por cinco estrategias propuestas en un marco para pertinencia cultural.

Resultados

El análisis interseccional permitió entender las sinergias y brechas de los esfuerzos y los protocolos que se implementan actualmente, tanto por parte de las comadronas como por el Ministerio de Salud. Encontramos que los servicios que actualmente se prestan para las cuatro enfermedades son en su mayoría independientes entre sí, y no se contaba con una estrategia integral de material educativo y de comunicación. Sin embargo, nuestros resultados se utilizarán como base para una estrategia de comunicación que sea coherente, localmente relevante y culturalmente apropiada para la población local, y también siga las regulaciones de las políticas nacionales.

Conclusiones

El uso de la interseccionalidad como método y como herramienta analítica nos permitió comprender (1) la interrelación de diversos determinantes sociales, culturales y ambientales que influyen en la prestación de servicios de salud, así como (2) la dinámica entre los sistemas de salud tradicional e institucional. (3) El compromiso de la comunidad y la participación de las diferentes partes interesadas en un proceso consultivo han sido fundamentales para los principios conceptuales y metodológicos de esta investigación. (4) Por último, otorgar un papel más destacado a las comadronas puede reforzar la sostenibilidad y la adecuación cultural, que es complementaria a la prestación de servicios de salud institucionales.

Palabras clave

Guatemala, comadronas, Chagas, sífilis, hepatitis B, VIH, interseccionalidad, enfermedades ETMI.

Antecedentes

Durante las últimas dos décadas, la Organización Panamericana de la Salud (OPS) ha promovido acciones para eliminar las enfermedades de transmisión materno infantil en las Américas, con un enfoque inicial en el VIH (virus de inmunodeficiencia humana) y la sífilis. En 2016, se incluyeron dos enfermedades adicionales, la hepatitis B y el Chagas, como se describe en el "Marco para la eliminación de la transmisión materno infantil del VIH, la sífilis, la hepatitis B y el Chagas" o ETMI Plus. La enfermedad de Chagas, aunque se encuentra principalmente en América Latina, con 70 millones de personas en riesgo de infección y causando 546,000 años de vida ajustados por discapacidad (AVAD) [1,2], ahora también se puede encontrar en el hemisferio norte debido a la migración. Sin embargo, dado que el vector no se encuentra en muchos países del norte [3], la principal preocupación son las transfusiones y la atención materno-infantil, ya que la seroprevalencia de individuos procedentes de países endémicos de la enfermedad de Chagas puede alcanzar el 18% [4]. En Guatemala, todavía existen algunas áreas con alta transmisión de la enfermedad con una seroprevalencia en mujeres en edad fértil del 10,5% [5].

En este sentido, Guatemala como estado miembro de la OPS, ha asumido el compromiso de mejorar la cobertura y el acceso a los servicios de planificación familiar para mujeres en edad reproductiva, incluyendo la atención prenatal, parto y postnatal, los programas de tamizaje, vacunación y tratamiento para la ETMI [6]. En 2019, el Programa Nacional de ETS (enfermedades de transmisión sexual), VIH y SIDA (síndrome de inmunodeficiencia adquirida, y síndrome de inmunodeficiencia adquirida respectivamente) de Guatemala informó que se realizaron pruebas de VIH, sífilis y hepatitis B a mujeres embarazadas con al menos un control prenatal. Las pruebas realizadas en el país indicaron que el 0,82% de las mujeres dieron positivo por VIH, el 0,21% por sífilis y el 0,19% por hepatitis B [7].

Las políticas nacionales en Guatemala aún no han alcanzado los objetivos establecidos por la OPS [8]. Sostenemos que las iniciativas locales que se basan en la participación de la comunidad son esenciales para la aplicación de las directrices de ETMI Plus mediante un enfoque culturalmente apropiado. En las regiones rurales y étnicamente diversas de Guatemala, donde el sistema de salud suele carecer de fondos suficientes, las comadronas locales son agentes clave en la prestación de atención a la salud materno infantil a nivel comunitario [9,10]. La contribución de las comadronas al sistema nacional de salud está oficialmente reconocida e incorporada en las directrices formales implementadas por el Ministerio de Salud Pública y Asistencia Social (MSPAS) [11], donde las comadronas son cada vez más reconocidas como actores importantes y partes interesadas en la prestación de atención sanitaria global, que va más allá de la salud de mujeres y niños. Las comadronas tienen el potencial de ser actores relevantes en los objetivos de ETMI Plus, especialmente en el papel que desempeñan en la detección, tratamiento y seguimiento de pacientes con VIH, sífilis, hepatitis B y Chagas. En general, el papel de las comadronas

proporciona puntos de articulación culturalmente apropiados y sostenibles entre el sistema de salud y la comunidad, agilizando la aplicación de programas de detección y tratamiento, especialmente en zonas rurales y remotas [7]. Sin embargo, las comadronas -especialmente las que articulan diferentes sistemas de conocimiento- tienden a quedar excluidas y marginadas del sistema de salud general [12]. Tomamos a las comadronas como punto central de nuestro análisis para considerar cómo la interseccionalidad es crucial en el desarrollo de programas culturalmente dirigidos y adaptados que tengan en cuenta la participación de la comunidad desde su inicio.

En 2019, nuestro equipo multidisciplinario ha llevado a cabo un proyecto de investigación para fortalecer el marco de ETMI Plus en Guatemala en el municipio de Comapa, Jutiapa, un contexto donde la atención perinatal, las desigualdades de género y las estructuras de poder emergen como determinantes sociales de la salud (DSS) interrelacionados [13]. El proyecto ha consistido en fases subsecuentes necesarias para llevar a cabo un programa adaptado al contexto, diseñado con un enfoque interseccional, que culminará con la producción de material educativo y comunicacional culturalmente apropiado para la promoción del tamizaje y tratamiento de las enfermedades ETMI Plus [14]. El objetivo de este proyecto es incorporar un enfoque interseccional de género en la relación entre el sistema de salud local y las comadronas, a través de un proceso colaborativo de elaboración de material educativo sobre enfermedades de transmisión vertical. El resultado deseado es fortalecer el sistema de salud pública reforzando las prácticas de salud preventivas en relación con las enfermedades de transmisión vertical.

Marco teórico

Los determinantes sociales de la salud (DSS) son definidos por la Organización Mundial de la Salud como "factores no médicos que influyen en los resultados de salud" [15] e incluyen una amplia gama de factores sociales relacionados con el sistema que crean las condiciones en las que las poblaciones o los individuos nacen, crecen, viven y trabajan. Los DSS pueden estar vinculados a condiciones individuales, sociales y estructurales [13] e influir directamente en las desigualdades producidas en el acceso a la atención de salud y en los resultados de la enfermedad para diferentes poblaciones e individuos.

La investigación relacionada con ETMI Plus ha tenido en cuenta los DSS, como las condiciones socioeconómicas y educativas de los individuos y las poblaciones y su acceso a servicios de salud accesibles y dignos [13,16]. La salud materno infantil se ve influida por normas construidas socialmente, "roles, comportamientos, actividades, atributos y oportunidades relacionados con el género" [13]. Por lo tanto, el género debe ser un DDS central para la prevención, la detección y el acceso al tratamiento de las enfermedades de transmisión materno infantil, ya que las desigualdades y las

relaciones de poder generadas por las normas sociales y estructurales de género repercuten en todos los niveles de la gestión de la enfermedad. Además, consideramos otros DSS, como las desigualdades socioeconómicas y la discriminación, las políticas institucionales y las condiciones que pueden influir en la consecución de los objetivos y metas de ETMI Plus [13,16]. Nuestro proyecto de investigación ha considerado los DSS a lo largo de todo el proceso de diseño e implementación, aplicando una estrategia interseccional con un enfoque de género (Figura 1), para lo cual seguimos una combinación de los enfoques de Kreuter et al. (2003), Torres-Ruiz et al. (2018), Campbell et al. (2013) y Ghasemi et al. (2021) [17–20].

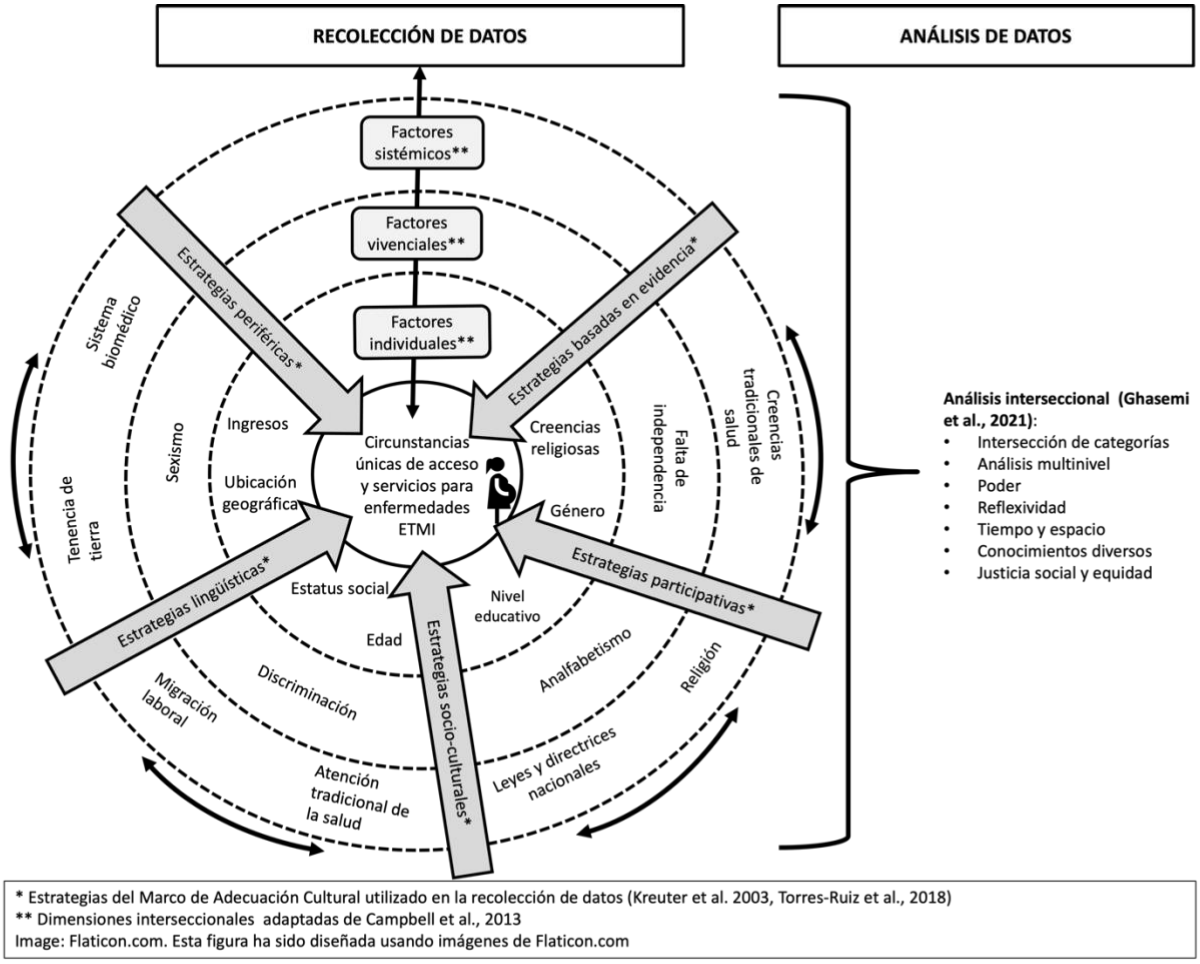

Figura 1. Marco teórico centrado en la persona [17–20]

La interseccionalidad puede entenderse como un método, una herramienta analítica y heurística [21,22] que ayuda a comprender “cómo los diferentes estratificadores sociales interactúan para crear diferentes experiencias de privilegio, vulnerabilidad y/o marginación” [13]. La interseccionalidad ha servido para abordar cuestiones relacionadas con el derecho, el feminismo, la teoría antirracista y la política. Sin embargo, cada vez es más habitual que académicos y activistas utilicen este concepto para abordar una amplia variedad de cuestiones relacionadas con identidades sociales, dinámicas de poder y sistemas

jurídicos y políticos. La característica multidimensional intrínseca de la interseccionalidad ha motivado su aplicación en distintas disciplinas [23]. El análisis de la interseccionalidad complementa el concepto de DSS al centrarse en "cómo las múltiples identidades sociales a nivel de experiencia individual (es decir, el micronivel) se entrecruzan con las desigualdades sociales multinivel al nivel macroestructural" [24]. Algunos estudios anteriores han aplicado la interseccionalidad de género y salud para comprender cómo abordar las diferencias en el acceso a una atención de salud accesible e integral [16,25], comprender la dinámica de poder y, el papel de los múltiples actores implicados en la atención de salud materno-infantil [20,26]. La interseccionalidad como metodología también se ha aplicado para comprender la conexión entre la adaptación cultural y el compromiso del paciente para mejorar los resultados de las poblaciones en riesgo de desigualdades [19].

Materiales y métodos

Este proyecto de investigación es resultado de la colaboración entre el Ministerio de Salud Pública y Asistencia Social de Guatemala (MSPAS), la Universidad del Valle de Guatemala (UVG) y la ONG internacional Fundación Mundo Sano. El equipo de investigación multidisciplinario -formado por antropólogos, biólogos, especialistas en salud pública, médicos, epidemiólogos y personal de salud de la zona de estudio (es decir, enfermeras, personal de control de vectores, comadronas)- permitió comprender de forma exhaustiva el problema de las enfermedades de transmisión vertical.

Sitio del estudio

Este trabajo se realizó en Comapa, municipio del departamento de Jutiapa, ubicado al sureste de Guatemala (Figura 2). El censo de 2018 registró 32,207 habitantes [27]. Debido a su frontera con El Salvador, el comercio y la migración son procesos clave que impactan la dinámica socioeconómica en Comapa. El contexto geográfico también influye en el comportamiento de búsqueda de atención médica, ya que algunas personas prefieren buscar tratamiento médico en El Salvador, dadas las condiciones de las carreteras y la proximidad a los centros de salud locales. El trabajo de larga data de varias instituciones nacionales e internacionales en Comapa ha buscado el manejo integral de la enfermedad de Chagas, que continúa con niveles de infestación del 15% a pesar de décadas de esfuerzos por controlar y eliminar la transmisión vectorial de esta zona [5,28–30]. Sin embargo, los avances logrados hasta la fecha en el control de la transmisión vectorial [31] nos han permitido ampliar nuestro trabajo y centrarnos en la transmisión congénita.

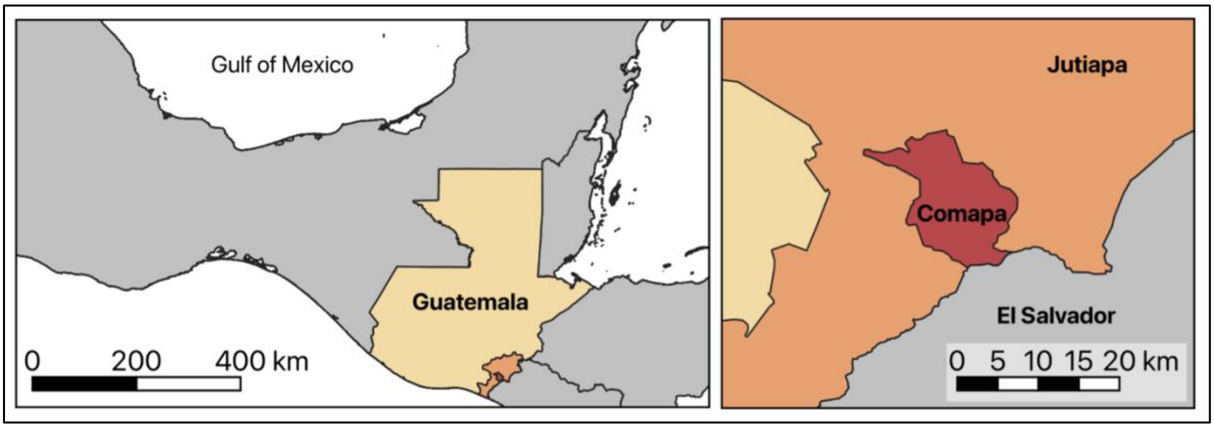

Figura 2. Ubicación geográfica de Comapa, Jutiapa, Guatemala. Mapa desarrollado en QGIS 3.26.

Población del estudio

El municipio de Comapa es diferente del Altiplano Maya, en el occidente del país, donde una proporción importante de la población se autoidentifica como indígena. Sin embargo, Comapa es étnicamente diversa: 0,7% de mayas, 0,2% de garífunas, 11,3% de xinkas, 0,7% de afrodescendientes, 87,1% de ladinos y menos del 0,1% de extranjeros [27]. Esto implica a menudo múltiples sistemas de conocimiento, plurilingüismo y tensas relaciones sociopolíticas en torno a los derechos sobre la tierra. Las relaciones dentro de la comunidad y entre ésta y el sistema de salud se ven influidas por esta compleja configuración étnica. Este proyecto se basa en un trabajo participativo previo realizado en la zona desde 2015 por un equipo multidisciplinario de la UVG que se centró en reforzar el tamizaje de la enfermedad congénita de Chagas [14]. La participación de la comunidad -un principio básico de dicha investigación- ha fomentado una fuerte relación con las comadronas locales, el personal de salud del MSPAS y otras organizaciones como las ONG internacionales que trabajan en Comapa.

Recolección de datos

Utilizamos diversos métodos de recolección de datos en 2019, con la participación de comadronas, personal del Ministerio de Salud Pública y Asistencia Social tanto a nivel nacional como local, y colaboradores externos (es decir, actores de otras instituciones que trabajan en temas relacionados con la salud materno-infantil en Comapa). Adicionalmente, se revisaron bases de datos oficiales con registros de tamizaje. El proceso de recolección de datos se basó en cinco estrategias del enfoque de adecuación cultural: A) estrategias basadas en la evidencia, B) estrategias de participación, C) estrategias socioculturales, D) estrategias lingüísticas y E) estrategias periféricas, siguiendo a Kreuter et al. (2003) y Torres-Ruiz et al. (2018) (Figura 3) [17,19]. La adopción de estas estrategias pretendía abordar las complejidades que surgen en las estrategias de salud pública en relación con las necesidades materno infantiles en este contexto rural, mejorando el éxito y la sostenibilidad de las intervenciones de salud a nivel comunitario [17].

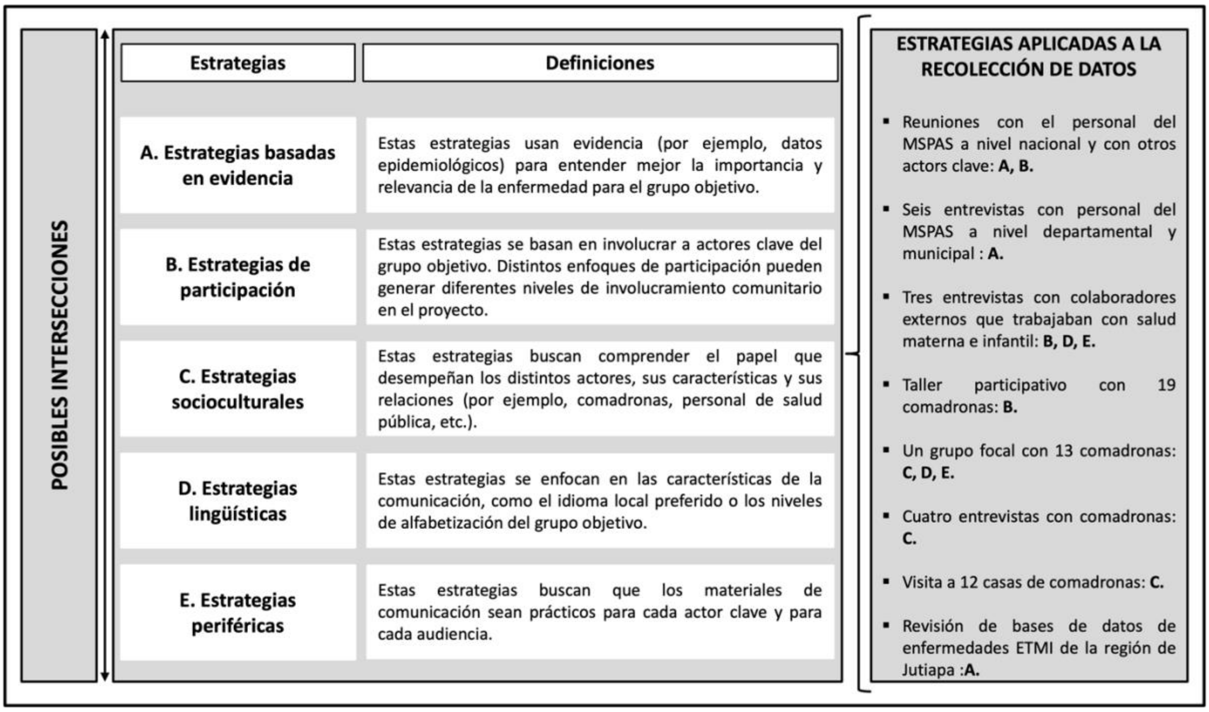

Figura 3: Estrategias del enfoque de adecuación cultural [17,19]

Dado que utilizamos varios métodos de recopilación de datos y que las cinco estrategias del marco de adecuación cultural no son mutuamente excluyentes, proporcionamos primero detalles sobre la selección de la población para explicar después cómo se aplicaron las estrategias durante la recopilación de datos (Figura 3). Las comadronas que participaron en las entrevistas y las actividades participativas se seleccionaron mediante una estrategia de muestreo intencional basada en criterios de alta asistencia a los talleres de formación impartidos y requeridos por el Ministerio de Salud Pública y Asistencia Social. Además, utilizamos la referencia en cadena para acompañar a un subconjunto de doce comadronas a sus hogares para realizar observación participante [32,33]. También utilizamos una estrategia intencionada para entrevistar al personal del MSPAS y a los “colaboradores externos”, basada en un muestreo por criterios, centrándonos en el personal que había colaborado anteriormente con el proyecto universitario y tenía un papel clave relacionado con la aplicación de las estrategias de ETMI Plus. Este grupo incluía enfermeras, médicos, personal de control de vectores y personal de una ONG. El personal del programa de control vectorial del Chagas tiene acceso a los hogares de las personas y conoce a la comunidad, lo que los convierte en actores importantes en la región [32,33].

Las entrevistas y los grupos de discusión se grabaron. Las grabaciones de audio fueron gestionadas por las antropólogas, que escucharon las grabaciones para hacer observaciones puntuales y profundizar en las entrevistas y la información que se iba a recopilar [32]. Con los datos cualitativos obtenidos en las entrevistas individuales y de grupo se buscó un punto de saturación teórica [32]. Todas las comadronas

y enfermeras del centro de salud local eran mujeres, y el equipo de investigación de la universidad estaba compuesto en su mayoría por investigadoras. El equipo de la ONG internacional estaba formado por dos hombres y una mujer. Las entrevistas y los grupos focales fueron realizados por dos antropólogas para reducir la dinámica jerárquica de género que puede surgir entre hombres y mujeres, que además pertenecen a grupos socioculturales diferentes.

Estrategias basadas en evidencia

Se consideraron los datos que contenían información de tamizaje de los pacientes a quienes se les había realizado la prueba de detección de alguna de las enfermedades ETMI Plus en el centro de salud local de Comapa y cuyas pruebas habían sido enviadas para confirmación al laboratorio de Jutiapa entre 2012 y 2019. En el laboratorio de Jutiapa se conservaban las bases de datos con los nacimientos registrados en cada municipio, incluido Comapa, y se presentaban datos no identificables de los pacientes que se habían sometido a las pruebas de VIH, sífilis, hepatitis B y Chagas. La información estaba desagregada por sexo, edad, comunidad de origen, lugar donde se realizó la prueba, fecha y resultado. Con esta información se obtuvo un panorama de cuántas pruebas relacionadas con la ETMI Plus se realizan en el centro de salud local de Comapa y se tiene una idea general de la incidencia de estas enfermedades en el municipio (Tabla 2).

De manera complementaria, para identificar las rutas de prevención, diagnóstico y tratamiento de las enfermedades ETMI Plus en Comapa, se realizaron seis entrevistas a personal de salud pública a nivel departamental y municipal (Hospital de Jutiapa: 2; Centro de Salud Comapa: 4).

Estrategias de participación

Consideramos la participación de los actores de las macroestructuras y las estructuras comunitarias relativas a la ETMI Plus; y las necesidades y expectativas de los actores locales que trabajan con la atención de salud materno infantil, incluidas las comadronas. Para identificar las macroestructuras y las estructuras comunitarias relevantes para el diagnóstico y el tratamiento de ETMI Plus, realizamos tres entrevistas adicionales con "colaboradores externos", que se identificaron porque ya trabajaban con la salud materno infantil en la zona y podían apoyar la sostenibilidad y la aceptabilidad de nuestros esfuerzos (véase la figura 3).

Las necesidades y expectativas de los actores locales proporcionaron material crucial para desarrollar un sólido enfoque de participación comunitaria. Se realizó un taller participativo en el centro de salud local de Comapa, donde diecinueve comadronas debatieron la transmisión congénita del VIH, la sífilis, la hepatitis B y la enfermedad de Chagas. Los objetivos del taller con las comadronas se centraron en generar conciencia sobre las enfermedades de transmisión vertical y comprender mejor las percepciones

de salud y enfermedad entre ellas. Este taller también proporcionó un espacio de diálogo para conocer las necesidades y expectativas relacionadas con las enfermedades de transmisión vertical. Esta última actividad también se consideró parte de las estrategias sociocultural, lingüística y periférica.

Estrategias socioculturales

A través de la observación participante, doce casas de comadronas fueron visitadas en múltiples ocasiones por el equipo de antropólogas, con el objetivo de comprender mejor y complementar la información generada con los otros métodos. Estas observaciones se contextualizaron dentro de trabajos previos realizados, ya que nuestro equipo de investigación tiene una presencia de larga data en la región [14,34]. Aunque nuestro objetivo era acompañar a las comadronas en sus consultas con las mujeres embarazadas, esto no fue posible debido a limitaciones de tiempo y recursos. Sin embargo, cuando las comadronas visitan a sus pacientes, suelen recordar a las mujeres sus citas y comprobar su bienestar, lo que forma parte de su atención rutinaria.

Se realizaron cuatro entrevistas en profundidad a comadronas seleccionadas en sus casas, un espacio en el que podían sentirse cómodas compartiendo sus experiencias. Estas entrevistas fueron realizadas en español por una de las antropólogas. El objetivo de estas entrevistas semiestructuradas era comprender cómo perciben las comadronas la salud y la enfermedad y su papel dentro del sistema de salud. Además, para complementar la información recolectada en las entrevistas, realizamos un grupo focal con trece comadronas del centro de salud local en una de las reuniones rutinarias de formación. Por último, realizamos tres entrevistas con colaboradores externos de otras instituciones relevantes identificadas en la fase anterior como estrechamente implicadas con la comunidad y que trabajan con madres y niños.

Estrategias lingüísticas y periféricas

La lengua materna de los participantes era el español, y los investigadores eran hispanohablantes nativas y habían realizado trabajo de campo en la región, por lo que comprendían las sutilezas del idioma. La mayoría de los participantes tenían conocimientos muy básicos de lectura y escritura; algunos sólo sabían firmar con su nombre. Esto significaba que todas las entrevistas, comunicaciones y grupos de discusión tenían que evitar los términos técnicos y permitir múltiples repeticiones de conceptos. A partir de los datos generados en la actividad del taller participativo, incluidos los dibujos que reflejan la comprensión de las comadronas de los conceptos clave, el equipo multidisciplinario está elaborando material educativo y de comunicación que refleje la perspectiva de la comunidad (una visión emic) sobre la prevención de la enfermedad ETMI.

Una de las principales preocupaciones del proceso de investigación ha sido garantizar la posterior aceptabilidad de la estrategia de comunicación [14]. Por último, las entrevistas llevadas a cabo con colaboradores externos nos ayudaron a triangular la información para identificar si todas las

instituciones que trabajan con la salud materna están utilizando los mismos mensajes y códigos lingüísticos.

Análisis de datos

El complejo análisis sociocultural llevado a cabo para el desarrollo de este proyecto de investigación puede desglosarse en las categorías propuestas por Ghasemi et al. (2021) en su revisión general sobre la interseccionalidad aplicada a las intervenciones de salud: categorías interseccionales, análisis multinivel, relaciones de poder, reflexividad, tiempo y espacio de la aplicación, conocimientos diversos y justicia social y equidad (Tabla 1) [18]. Dentro de estas categorías generales, los autores enumeran múltiples preguntas orientativas diseñadas para servir de indicaciones generales que garanticen la interseccionalidad de un programa en las fases de identificación, diseño/aplicación y evaluación. Reunimos los datos recolectados de todas las fuentes, reflexionando sobre cada una de las categorías y utilizando las preguntas para guiar el análisis. Las entrevistas y las actividades de los grupos focales con comadronas se clasificaron en cuatro temas generales: (1) centro/sistema de salud, (2) papel social de las comadronas, (3) naturaleza y (4) salud y enfermedad. Además, otras formas de datos, como los mapas de actores, los datos epidemiológicos y las revisiones bibliográficas, también pueden organizarse en estas categorías, que a menudo se solapan. Por lo tanto, es crucial comprender la interacción entre las distintas categorías para apreciar toda la complejidad de la situación. En este documento, nos centramos en las dos primeras fases, ya que la tercera fase de la evaluación aún no se ha completado.

CATEGORY	GUIDING QUESTIONS
**Categorías interseccionales**	¿Se ha tenido en cuenta la combinación de diferentes factores sociales, como la edad, el sexo, la raza/etnia, la clase social y la migración, a la hora de identificar las causas del problema?
**Análisis multinivel**	¿Se han tenido en cuenta los diversos factores a nivel individual, interpersonal, organizativo y de gobernanza en el proceso de identificación de problemas? ¿Se han tenido en cuenta las intersecciones de los factores sociales en los niveles micro, meso y macro?
**Relaciones de poder**	¿Han participado las partes interesadas, como las poblaciones afectadas, en la identificación del problema? ¿Se han abordado las estructuras de poder, como las políticas y las leyes, responsables del encuadre del problema de salud? ¿Se ha enmarcado la intervención/el programa en el contexto cultural, político, económico y social actual? ¿Y ha reflejado las necesidades de las poblaciones afectadas?

**Reflexividad**	¿El comité de planificación/equipo de investigación examina de forma crítica sus valores, experiencias, creencias y suposiciones sobre el problema de salud? ¿Tienen los investigadores/planificadores de salud una práctica reflexiva?
**Tiempo y espacio de la aplicación**	¿Se ha tenido en cuenta el proceso de planteamiento del problema a lo largo del tiempo (históricamente) o en diferentes lugares (geográficamente) y los cambios de privilegios y desventajas, incluidas las identidades que se entrecruzan y los procesos que determinan su valor a lo largo del tiempo y el lugar? ¿Es la intervención/el programa flexible en cuanto a las condiciones de tiempo y lugar?
**Conocimientos diversos**	¿Se ha utilizado la perspectiva de las personas habitualmente marginadas en el proceso de identificación del problema? ¿Se han generado los conocimientos a partir de varios recursos, como la investigación cualitativa o cuantitativa, los datos empíricos o interpretativos y los conocimientos indígenas? ¿Se han utilizado los conocimientos del grupo destinatario en el proceso de diseño y ejecución del programa de salud?
**Justicia social y equidad**	¿Se centran las intervenciones/programas actuales en la promoción de la salud de los grupos vulnerables? ¿Se ha diseñado y aplicado la intervención/el programa para reducir las desigualdades?

Tabla 1: Categorías para el análisis interseccional con algunas preguntas aplicadas en nuestro proceso de análisis. Adaptado de Ghasemi et al. (2021) [18]

Garantía de calidad

Hemos añadido como archivo adicional (archivo adicional 1) la lista de comprobación de los Criterios consolidados para la presentación de informes de estudios cualitativos (COREQ) [35], que incluye detalles relevantes sobre el equipo y el proceso de investigación. Durante la recopilación y el análisis de datos, triangulamos [36] entre métodos y participantes. La inclusión de esta estrategia mejoró los esfuerzos de interseccionalidad, ya que las perspectivas son complementarias y nos permiten obtener una visión más amplia de la situación. A lo largo de todo el proceso de recolección y análisis de datos, aplicamos el intercambio de información entre los investigadores y los colaboradores del Ministerio de Salud Pública y Asistencia Social [32,36].

Resultados

Las observaciones, entrevistas, grupos focales, talleres y revisiones bibliográficas han arrojado una imagen compleja de la situación de la iniciativa ETMI Plus en Comapa. La relación entre los actores revela el funcionamiento del sistema de salud. Los factores sociopolíticos e históricos son cruciales para comprender el papel social actual de las comadronas en su contexto. Argumentamos que la comprensión

de las interacciones entre los diferentes sistemas de conocimiento (y los significados de salud, enfermedad y naturaleza) determina el éxito de las políticas de salud pública sobre la prevención, diagnóstico y tratamiento para la ETMI Plus y la salud materno-infantil en general.

Sistema de salud

Mapa de actores de múltiples niveles que configuran el sistema de salud

Desarrollamos un mapa de actores (Figura 4), crucial para los procedimientos posteriores, para garantizar la participación transversal en la aplicación de las políticas de salud (prevención, diagnóstico, tratamiento) relacionadas con la ETMI Plus.

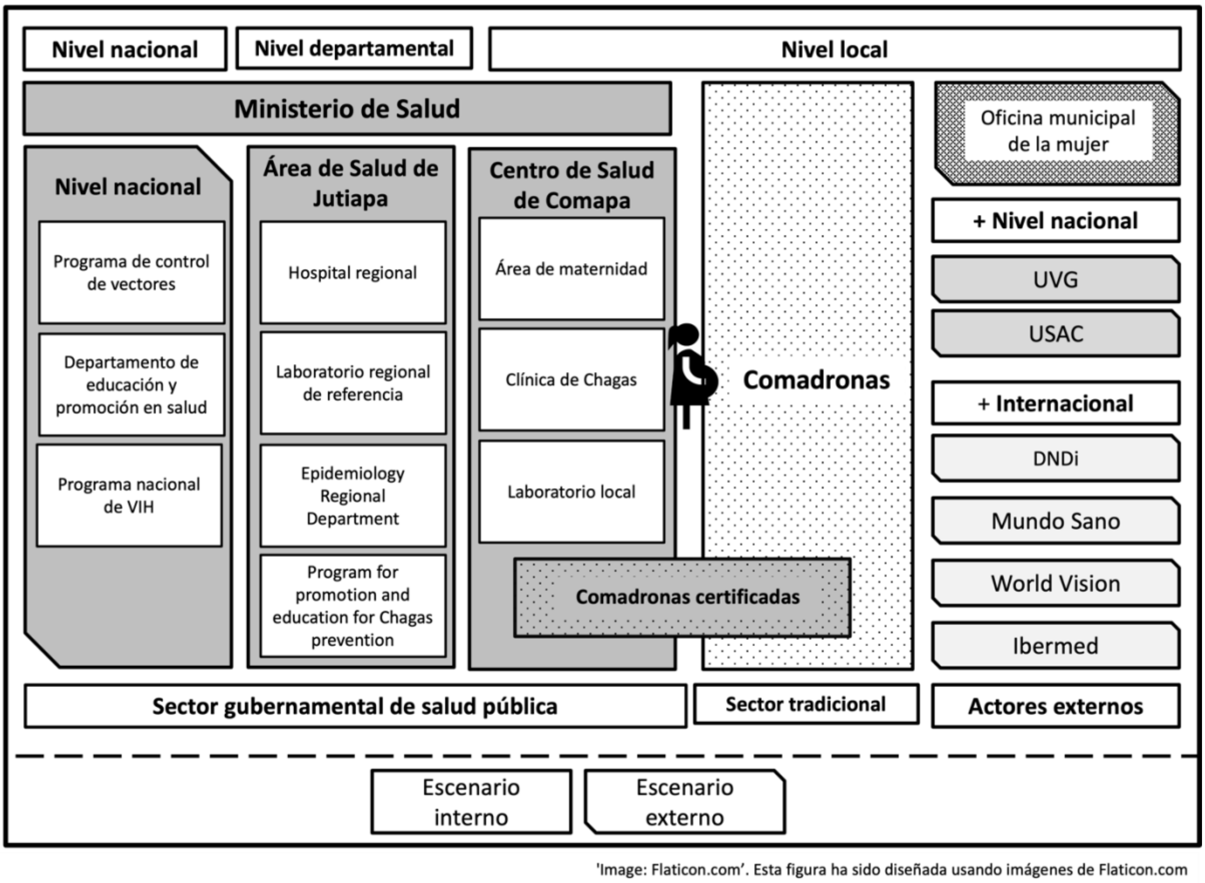

Figura 4. Mapa de actores. Esta figura muestra las instituciones y actores implicados en la atención perinatal a nivel local, departamental y nacional. La figura muestra la dicotomía entre el sector público de salud y el sector tradicional (comadronas), así como los factores externos ("entorno externo") identificados a nivel local, en los que se centró nuestra investigación. *USAC es Universidad de San Carlos de Guatemala, UVG es Universidad del Valle de Guatemala y DNDi es Iniciativa de Medicamentos para Enfermedades Desatendidas. Aunque no es exhaustivo, este mapa muestra las partes interesadas identificadas en el momento del estudio y los posibles colaboradores

La principal parte interesada que determina el contenido y la dirección de las iniciativas relacionadas con la salud en Guatemala es el Ministerio de Salud Pública y Asistencia Social. Para el caso de las enfermedades de la iniciativa ETMI Plus, el Ministerio de Salud ha desarrollado programas de prevención, diagnóstico y tratamiento que abarcan el control de vectores, las iniciativas epidemiológicas y la educación y promoción de la salud a nivel nacional, regional y local. Estos programas son ejecutados a nivel departamental y local por los centros de salud regionales y locales (es decir, hospitales regionales, laboratorios de referencia, departamentos de epidemiología y programas locales de gestión de Chagas). A nivel comunitario muy local, la Clínica de Chagas, las salas de maternidad, las comadronas y los laboratorios locales son responsables de aplicar las directrices nacionales y regionales.

Además, actuando a nivel local, nacional e internacional, es posible encontrar organizaciones no gubernamentales que asumen funciones de salud pública siguiendo una agenda personal o global (como DNDi, Visión Mundial, Fundación Mundo Sano, Ibermed y otras). Los actores identificados en la Figura 4 contribuyen de distintas maneras a los esfuerzos de prevención, diagnóstico y tratamiento de las enfermedades de la ETMI Plus. La interacción de los distintos actores influye en la configuración de lo que se entiende por sistema público de salud, un concepto ambiguo y no fácil de definir, pero esencial en la creación y aplicación de políticas de salud.

Las comadronas como actores clave en la implementación de los programas de la ETMI Plus

En 2015, CARE International registró una media de 23,000 comadronas en Guatemala, 16,000 de las cuales habían recibido formación del sistema de salud oficial [37]. Estas comadronas atendieron dos tercios de todos los partos en Guatemala, el 80% de los cuales se produjeron en zonas rurales mayas [12]. Gran parte del trabajo centrado en las comadronas guatemaltecas se ha llevado a cabo en las zonas montañosas occidentales entre poblaciones que se identifican a sí mismas como mayas. Dado que la población del sureste de Guatemala tiene una autoidentificación menos definida con los grupos indígenas, los estudios sobre sistemas de salud alternativos tienden a dejar fuera a estas poblaciones, a excepción de los trabajos de Bossen (1984) y Cominsky (2016) [12,38].

Las comadronas que participaron en las entrevistas y grupos focales tenían aproximadamente 60 años, y muchas llevaban más de 10 años atendiendo a una media de cuatro mujeres al año. Sin embargo, no existe un perfil homogéneo de comadronas en Comapa. Todas tienen diferentes niveles de educación (no todas aprendieron a leer o escribir), y desempeñan diferentes funciones dentro de la comunidad en función de su participación en otros grupos como la iglesia, ONGs o el gobierno local. Algunas de ellas también practican otras formas de curación alternativa, ampliando sus conocimientos y su papel dentro de la comunidad.

Convertirse en comadrona en la región de Comapa no sigue la misma trayectoria que en las tierras altas mayas del oeste del país, donde las mujeres reciben su vocación a menudo en sueños [12]. Las comadronas en nuestra área de estudio han recibido capacitación de sus abuelas o incluso del centro de salud local, y no necesariamente obtienen su sustento únicamente de su trabajo de partería. Las mujeres a las que asisten les pagan un salario simbólico cuando es posible. A veces son seleccionadas por los líderes locales en el consejo comunitario de desarrollo (COCODE - Concejos Comunitarios de Desarrollo) por su capacidad de liderazgo [39].

No todas las comadronas de Comapa asisten a la formación impartida por el centro de salud local. En consecuencia, no todas pueden inscribir a los recién nacidos en el RENAP (Registro Nacional de las Personas). De igual forma, no todas cuentan con capacitación técnica para identificar riesgos y ofrecer recomendaciones para las mujeres embarazadas. Aunque las comadronas reciben información sobre la ETMI Plus durante la formación en los centros de salud locales, no están oficialmente capacitadas para examinar a las embarazadas ni a los recién nacidos en un entorno comunitario. Sin embargo, a menudo proporcionan información importante a sus pacientes y sus familias, principalmente sobre salud sexual y reproductiva. El centro de salud local de Comapa no comparte los resultados de las pruebas realizadas a las embarazadas y los recién nacidos con las comadronas, y cada mujer decide si comparte la información personalmente con su comadrona. Las comadronas que han recibido formación en el centro de salud local y acompañan a las mujeres embarazadas durante todo el embarazo y el posparto proporcionan apoyo emocional y comparten conocimientos tradicionales y algunos biomédicos. En el caso del Chagas en Comapa, hay múltiples organizaciones e instituciones implicadas, desde el control vectorial hasta el diagnóstico y el tratamiento, como se muestra en el mapa de actores (Figura 4). No todas las zonas de Guatemala presentan una red tan compleja de actores relacionados con el Chagas.

Diversas formas de conocimiento

Dado que en 2018, el 25 % de la población total mayor de 4 años no tenía ningún nivel de educación formal, existen altas tasas de analfabetismo en Comapa [27], y cualquier material educativo y de comunicación debe ser visualmente claro y reflejar los puntos de vista étnicos y de género locales. Además, las comadronas de Comapa manejan una combinación de sistemas de conocimiento en torno a la salud y el bienestar. Aunque no se identifican como mayas, la influencia de las creencias xinka ha calado en su forma de entender la salud, las relaciones sociales y las nociones de bienestar que van más allá de la conceptualización individualista biomédica de la salud y la curación [40]. La creencia maya de que un cuerpo debe estar en equilibrio dentro de sí mismo, así como con la comunidad y el entorno [41], y la importancia de equilibrar los estados frío-caliente dentro del cuerpo fueron conceptos evidentes que surgieron a través de las entrevistas con las comadronas de Comapa.

Las comadronas representaron sus concepciones de la salud y la curación a través de expresiones visuales. Éstas se clasificaron en las siguientes categorías: (1) naturaleza, (2) rol social y (3) el centro de salud local, de acuerdo con los temas que surgieron en las entrevistas. Los dibujos también se interpretaron en relación con las entrevistas y las conversaciones informales con las comadronas. Estas visiones emic de la salud y el bienestar destacan características que deben tenerse en cuenta en la elaboración de material educativo y de comunicación.

Naturaleza

La presencia destacada de la naturaleza en los dibujos -vinculada a la flora y la fauna- era explicada por las comadronas como un símbolo de vida. Además, las plantas también se utilizan para curar dolencias, ya que las comadronas reciben de sus abuelas conocimientos sobre las propiedades medicinales de las plantas. También se dibujaron animales, ya que constituyen una presencia importante en la vida cotidiana: los burros sirven de transporte en zonas remotas o se utilizan para labrar los campos, las gallinas proporcionan huevos, los cerdos carne, los gatos mantienen las casas libres de ratones que, de otro modo, se comerían las reservas de maíz (fundamental para la subsistencia y rico en valor simbólico).

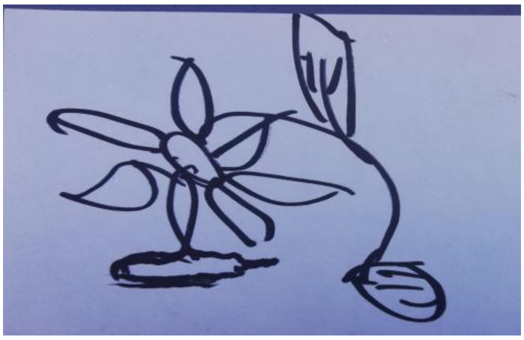

Imagen 1

"Dibujé a la salud como una hoja, a la mamá como la planta y al bebé como el botón." (Comadrona 1, 2019)

Rol Social

La categoría del mundo social se construyó a partir de los relatos de las comadronas sobre el impacto de sus actividades en las vidas de otras personas de su comunidad, lo que les otorgaba valor como sanadoras dentro de un contexto social.

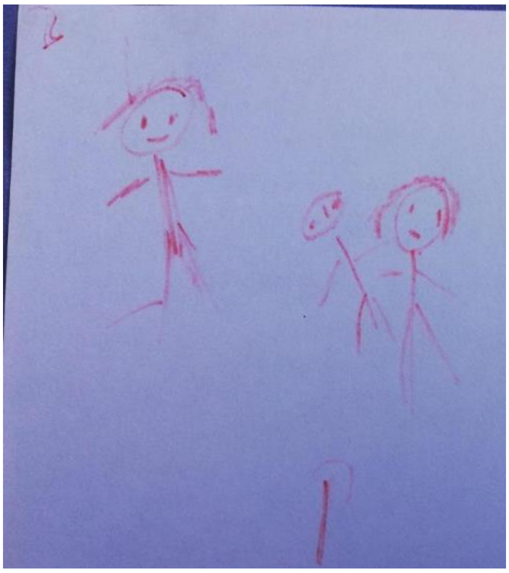

Imagen 2

"Estoy contenta con las personas que llegan a mi casa. Yo las ayudo. Cuando puedo pues si, y cuando no también..." (Comadrona 11, 2019).

Este comentario debe entenderse en el contexto de la importancia del apoyo y la empatía en el proceso de curación. La curación en las prácticas mayas tiene un gran componente relacional [41], que depende de que la familia inmediata, así como la comunidad en general y los antepasados, se impliquen en los procesos de curación. Por lo tanto, aunque las comadronas no tengan las herramientas o la formación necesarias para tratar directamente el problema de salud inmediato por el que se las consulta, actúan como facilitadoras y apoyo en el proceso de corrección del desequilibrio que provoca la enfermedad.

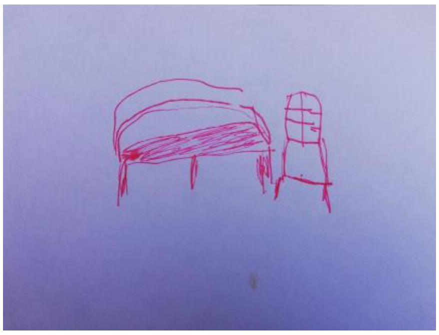

Imagen 3

"Yo no puedo dibujar ni leer, pero dibujé una silla y una cama. Yo también me siento alegre porque he dado vida a bastantes niños. Tengo niños de 23 años. Antes de capacitarme yo salía a ayudar, y tengo 15 años…" (Comadrona 12, 2019).

El Centro de Salud Local

Aunque a veces las comadronas consiguen material básico de primeros auxilios en el centro de salud local, sus principales "herramientas" son sus manos, con las que palpan el vientre de las embarazadas y la posición de los bebés antes del parto.

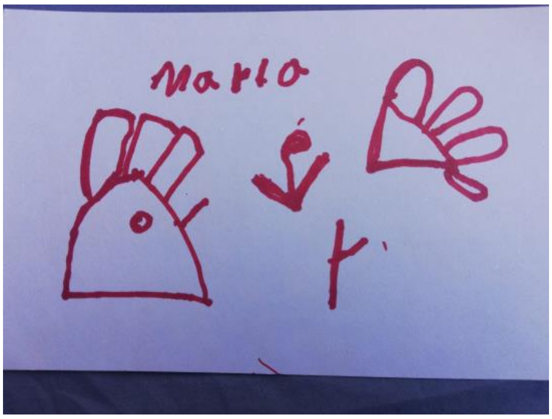

Imagen 4

“Yo dibujé mis manos porque mis manos hacen lo bueno en todas las personas que me buscan” (Comadrona 8, 2019).

El siguiente comentario, que no fue aislado, apunta a las jerarquías estructurales que se reproducen en las sesiones de formación que reciben las comadronas, en las que el mensaje más importante es la redirección de las mujeres al centro de salud local.

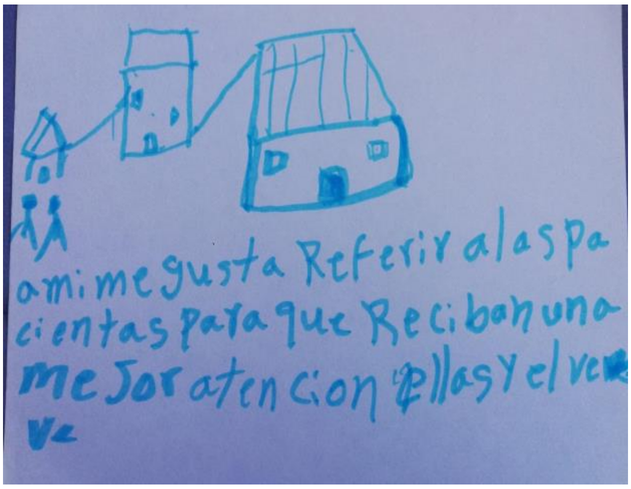

Imagen 5

“Yo dibujé una casita, la casa de la paciente y ahí referí al centro. Ya después no sé si la llevaron al hospital o no, pero yo referí. A mi lo que más me gusta es referir a las pacientes para que reciban una mejor atención, y el bebé” (Comadrona 4 - 2019).

Diagnóstico, prevención y tratamiento

A pesar de formar parte de una estrategia oficial integrada de la iniciativa ETMI Plus, estas cuatro enfermedades aún no se abordan mediante un protocolo unificado, aunque en la práctica existen muchas similitudes en la forma de abordarlas (Figura 5). En la fase de prevención, que se refiere principalmente a la educación y asistencia de las mujeres embarazadas al centro de salud local, el personal médico (médicos y enfermeras) y las comadronas trabajan conjuntamente para informar a las pacientes y dirigirlas al parto institucional. La etapa de diagnóstico está a cargo principalmente del centro de salud local, en el cual se realizan pruebas rápidas de tamizaje de las cuatro enfermedades en el laboratorio de Comapa, y las muestras positivas son enviadas al laboratorio de Jutiapa y posteriormente al laboratorio nacional para su confirmación.

En cuanto al tratamiento de pacientes positivos, la sífilis, la hepatitis B y el Chagas son tratados a nivel local en el centro de salud; sin embargo, los pacientes VIH positivos son referidos al Hospital de Jutiapa a nivel departamental. El tratamiento para VIH, sífilis y Chagas es gratuito, y la disponibilidad para los tratamientos de VIH y Chagas es proporcionada por el Ministerio de Salud y debe ser tramitada por el mismo. Mientras que la sífilis debe tratarse durante el embarazo para evitar malformaciones en el feto (y tanto la madre como el padre deben recibir tratamiento para evitar la reinfección), el tratamiento del Chagas no puede administrarse hasta después del parto y el periodo de lactancia. El seguimiento de las pacientes lo realizan conjuntamente las comadronas y el personal de los centros de salud locales. Se recomienda el parto institucional para las mujeres seropositivas para cualquiera de las cuatro enfermedades, siendo el VIH, la sífilis y la hepatitis B partos por cesárea en el hospital departamental, ya que todavía no están equipados para realizarlos a nivel local. El parto institucional también permite el seguimiento médico de los recién nacidos, que incluye una vacuna contra la hepatitis B.

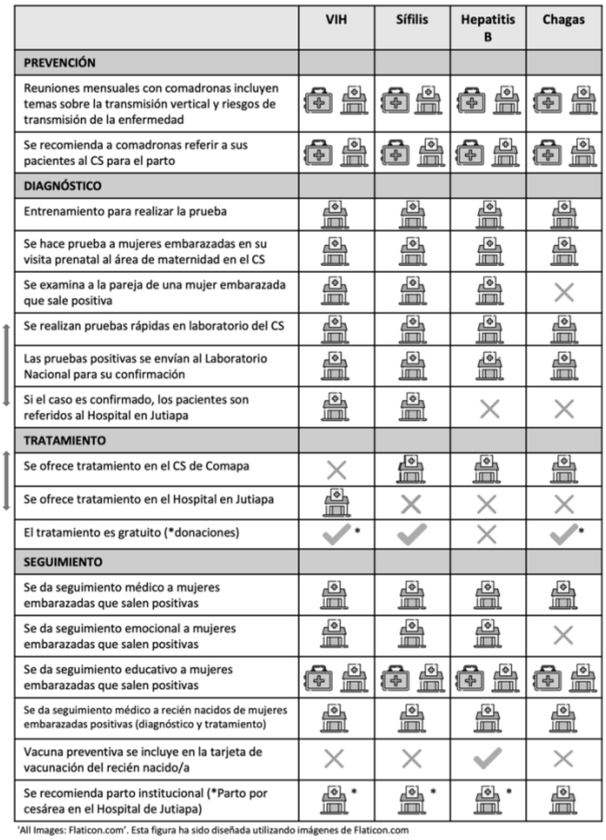

Figura 5 Comparación de las vías de prevención, diagnóstico, tratamiento y seguimiento de las cuatro enfermedades incluidas en la iniciativa ETMI Plus. La figura 5 muestra las actividades realizadas tanto por el centro de salud local de Comapa como por las comadronas. El cuadro está dividido según las principales actividades perinatales, que también representan algunas de las etapas clave de los objetivos de la ETMI Plus. El icono del botiquín representa la función de las comadronas, mientras que el icono de la clínica representa las funciones que desempeña el Ministerio de Salud en sus distintos niveles.

Los resultados compartidos por el laboratorio de Jutiapa (Tabla 2) señalan una diferencia entre las estrategias de tamizaje de las mujeres embarazadas en Comapa con respecto a todas las enfermedades de la ETMI Plus. Mientras que el tamizaje y seguimiento de estas enfermedades están oficialmente bajo la responsabilidad de los centros de salud locales, las comadronas están involucradas principalmente en las etapas de prevención y seguimiento. A veces, las comadronas acompañan a las pacientes y sus familias en las etapas previas al parto, durante el parto y después del parto. Aunque las comadronas han recibido formación en el centro de salud local para animar a sus pacientes a tener partos institucionales asistidos, muchas veces las pacientes no llegan a las instalaciones oficiales por emergencias, falta de tiempo, problemas de accesibilidad o decisiones personales. Los registros de partos y seguimientos de mujeres embarazadas que se llevan en el Laboratorio Regional del Hospital de Jutiapa sobre pruebas y partos registrados en Comapa mostraron que, aunque las mujeres acuden al centro de salud local para sus controles, muchas deciden seguir atendiendo su pre y posparto con las comadronas. Estas interacciones no son mutuamente excluyentes.

Datos epidemiológicos de: VIH, Sífilis, Hepatitis B, y la enfermedad de Chagas

Presentamos los datos compartidos por el laboratorio de Jutiapa entre 2012 y 2019, centrándonos específicamente en las muestras tomadas en Comapa, por ser el municipio donde se desarrolló nuestro trabajo. La enfermedad con más muestras tomadas para confirmación fue la enfermedad de Chagas, seguida de la sífilis y el VIH, con solo 14 muestras tomadas para hepatitis B.

Disease	CHAGAS n (%)			HEPATITIS B n (%)			SYPHILIS n (%)			HIV n (%)
Age range	F	M	Total	F	M	Total	F	M	Total	Total
Less than 1	2 (0.5)	1 (0.5)	3 (0.5)	0 (0.0)	0 (0.0)	0 (0.0)	0 (0.0)	0 (0.0)	0 (0.0)	0 (0.0)
1-4	2 (0.5)	2 (1.0)	4 (0.7)	0 (0.0)	0 (0.0)	0 (0.0)	0 (0.0)	0 (0.0)	0 (0.0)	0 (0.0)
5-9	23 (5.7)	34 (17.5)	57 (9.5)	0 (0.0)	0 (0.0)	0 (0.0)	0 (0.0)	0 (0.0)	0 (0.0)	0 (0.0)
10-14	69 (17.0)	69 (35.6)	138 (23.0)	0 (0.0)	0 (0.0)	0 (0.0)	0 (0.0)	0 (0.0)	0 (0.0)	0 (0.0)
15-19	37 (9.1)	28 (14.4)	65 (10.9)	2 (14.3)	0 (0.0)	2 (14.3)	3 (13.3)	0 (0.0)	3 (11.5)	2 (8.0)
20-24	50 (12.3)	6 (3.1)	56 (9.3)	4 (28.6)	0 (0.0)	4 (28.6)	2 (9.1)	2 (13.3)	4 (15.4)	6 (24.0)
25-29	61 (15.1)	12 (6.2)	73 (12.2)	3 (21.4)	0 (0.0)	3 (21.4)	1 (4.5)	0 (0.0)	1 (3.8)	8 (32.0)
30-34	44 (10.9)	6 (3.1)	50 (8.3)	2 (14.3)	0 (0.0)	2 (14.3)	2 (9.1)	0 (0.0)	2 (7.7)	2 (8.0)
35-39	37 (9.1)	7 (3.6)	44 (7.3)	1 (7.1)	0 (0.0)	1 (7.1)	2 (9.1)	1 (6.7)	3 (11.5)	4 (16.0)
40-44	38 (9.4)	4 (2.1)	42 (7.0)	2 (14.3)	0 (0.0)	2 (14.3)	8 (36.4)	0 (0.0)	8 (30.8)	2 (8.0)
45-49	7 (1.7)	4 (2.1)	11 (1.8)	0 (0.0)	0 (0.0)	0 (0.0)	0 (0.0)	0 (0.0)	0 (0.0)	1 (4.0)
50-54	13 (3.2)	3 (1.5)	16 (2.7)	0 (0.0)	0 (0.0)	0 (0.0)	1 (4.5)	0 (0.0)	1 (3.8)	0 (0.0)
55-59	7 (1.7)	5 (2.6)	12 (2.0)	0 (0.0)	0 (0.0)	0 (0.0)	1 (4.5)	0 (0.0)	1 (3.8)	0 (0.0)
60-64	11 (2.7)	3 (1.5)	14 (2.3)	0 (0.0)	0 (0.0)	0 (0.0)	2 (9.1)	0 (0.0)	2 (7.7)	0 (0.0)
65-69	3 (0.7)	5 (2.6)	8 (1.3)	0 (0.0)	0 (0.0)	0 (0.0)	0 (0.0)	0 (0.0)	0 (0.0)	0 (0.0)
70-74	0 (0.0)	3 (1.5)	3 (0.5)	0 (0.0)	0 (0.0)	0 (0.0)	0 (0.0)	0 (0.0)	0 (0.0)	0 (0.0)
75-79	0 (0.0)	1 (0.5)	1 (0.2)	0 (0.0)	0 (0.0)	0 (0.0)	0 (0.0)	1 (6.7)	1 (3.8)	0 (0.0)
80-84	0 (0.0)	0 (0.0)	0 (0.0)	0 (0.0)	0 (0.0)	0 (0.0)	0 (0.0)	0 (0.0)	0 (0.0)	0 (0.0)
85-89	0 (0.0)	1 (0.5)	1 (0.2)	0 (0.0)	0 (0.0)	0 (0.0)	0 (0.0)	0 (0.0)	0 (0.0)	0 (0.0)
90 y +	1 (0.2)	0 (0.0)	1 (0.2)	0 (0.0)	0 (0.0)	0 (0.0)	0 (0.0)	0 (0.0)	0 (0.0)	0 (0.0)
Total n (%)	405 (68)	194 (32)	599 (100)	14 (100)	0 (0.0)	14 (100)	22 (85)	5 (15)	26 (100)	25 (100)

Tabla 2. Total de muestras analizadas en el laboratorio de Jutiapa provenientes del centro de salud de Comapa entre 2012 y 2019 por enfermedad y sexo.

Al analizar los datos por sexo, la mayoría de las muestras para las cuatro enfermedades se obtuvieron de mujeres, lo que representa más de dos tercios del total de muestras tomadas para Chagas y sífilis.

Todas las muestras de hepatitis B procedían de mujeres. Los datos de los pacientes con VIH no se especificaron por sexo. El hecho de que los registros de las cuatro enfermedades sean mayoritariamente femeninos sugiere que los hombres no acuden a las pruebas con la misma frecuencia que las mujeres o que el sistema de salud no tiene en cuenta a los hombres en los protocolos de detección. La promoción del Ministerio de Salud Pública y Asistencia Social junto con la Universidad del Valle de Guatemala para el tamizaje materno-infantil de la enfermedad de Chagas ha sido persistente desde 2015 [14], lo que podría explicar la diferencia en la cantidad de hombres y mujeres tamizados en comparación con las otras tres enfermedades. Sin embargo, la inclusión de hombres sería importante, considerando que algunas de las enfermedades de la ETMI Plus son de transmisión sexual. Especialmente en el caso de la sífilis, la reinfección de las mujeres durante el embarazo puede provocar defectos congénitos en el bebé.

La mayoría de los resultados de las pruebas para las cuatro enfermedades se realizaron en personas de entre 15 y 50 años (edad reproductiva de la mujer). Además, el elevado número de hombres y mujeres de entre 10 y 14 años sometidos a pruebas de detección de Chagas puede corresponder a las actividades de detección realizadas en las escuelas. En cuanto a los datos de recién nacidos y niños, sólo se notificaron pruebas para la enfermedad de Chagas.

Discusión

Uno de los mayores retos en la aplicación de programas de salud es la complejidad de los conceptos de salud y enfermedad en contextos culturalmente diversos [42]. Por lo tanto, el éxito o el fracaso de una intervención depende en gran medida de su adecuación cultural, es decir, de su capacidad para considerar las particularidades del grupo destinatario, su aceptación dentro de la comunidad y su sostenibilidad [20,43].

El peso de los determinantes sociales de la salud en el diagnóstico, el tratamiento y la prevención de la ETMI Plus exige que se les preste atención desde el diseño y la aplicación en la estrategia de salud. Las características históricas, económicas, políticas e institucionales influyen en el grado de vulnerabilidad de una persona a la enfermedad. Esto implica "tener un conocimiento suficiente de los mecanismos que influyen en las desigualdades en salud y adoptar un marco conceptual que no sólo aclare la relación entre los determinantes sociales y las desigualdades en salud, sino que también ayude a identificar los puntos de entrada para la intervención" [44].

La multiplicidad de patrones culturales que se dan en la región aporta una complejidad adicional, que exige no sólo prestar atención a los determinantes estructurales generales de la salud, como la pobreza, el analfabetismo o la situación laboral, sino también a características culturales más matizadas, como

los conflictos por la tenencia de la tierra, las nociones alternativas de salud y enfermedad y los diferentes patrones de parentesco [45]. El enfoque específico y adaptado de los programas de promoción de la salud descrito por Kreuter et al. (2003) propone intervenciones adaptadas a “un subgrupo de población definido que tiene en cuenta las características compartidas por los miembros del subgrupo” y hace uso de cinco estrategias para llevar a cabo un programa de forma culturalmente adaptada [17,25,46]. Estas estrategias (mencionadas en la figura 2) ayudan a abordar las intersecciones, la complejidad y la heterogeneidad de las personas de una comunidad en función de factores individuales, experiencias y su relación con el sistema más amplio [20]. También generan un enfoque más interseccional al exigir interacciones de conocimientos entre distintos sectores y disciplinas y un proceso consultivo con todos los actores [25].

Recordando que la interseccionalidad puede servir como un método - descrito anteriormente - así como una herramienta analítica [21,46], ahora nos centramos en este segundo aspecto del concepto mediante la identificación de las categorías destacadas por Ghasemi. et al. (2021) que emergen de nuestro diseño e implementación del proyecto: categorías interseccionales, análisis multinivel, relaciones de poder, reflexividad, tiempo y espacio de la aplicación, conocimientos diversos, y justicia social y equidad [18,20].

El contexto cultural e histórico del presente proyecto han sido el punto de partida de la agenda de investigación del equipo multidisciplinario. El proceso de recopilación de datos captó los antecedentes históricos y las características culturales de los habitantes locales de Comapa, elaborados mediante la consulta de material de archivo y la investigación de campo, y demostró las categorías sociales, económicas y políticas que configuran las experiencias de salud en la región. Dado que las categorías de estratificación social (etnia, género, raza, estatus social) están estrechamente vinculadas a otros determinantes de la salud (económicos, ambientales), un análisis interseccional revela cómo los diferentes estratificadores sociales interactúan entre sí para condicionar el acceso, la prevención, el diagnóstico y la gestión de la salud [19,20,25].

Al centrarnos en las comadronas y las enfermedades de transmisión vertical, los participantes en nuestra investigación fueron principalmente mujeres. Trabajar en colaboración con las comadronas locales es imprescindible para promover la igualdad étnica y de género mediante el desarrollo de material educativo y de comunicación culturalmente apropiado. Sin embargo, dadas las estructuras patriarcales de las comunidades rurales de Comapa, nos encontramos a menudo con que teníamos que hablar con hombres y necesitábamos que nos acompañara en el trabajo de campo un personal técnico masculino, ya que éramos mayoritariamente investigadoras. Así pues, desde el inicio del proyecto de investigación desgranamos las categorías de género [20,45].

La presencia masculina en los procesos relacionados con la salud (concretamente en los procesos de la ETMI Plus) es una cuestión compleja. Las comadronas entrevistadas mencionaron prohibiciones impuestas por los maridos a sus mujeres relacionadas con la gestión del embarazo: control de la natalidad, capacidad para asistir a los controles del embarazo en el centro de salud local, imposibilidad de acudir al hospital sin el patrocinio masculino y falta de permiso de las comadronas para ver a las embarazadas sin el consentimiento o la presencia del marido. Para las mujeres, negarse a cumplir este tipo de prohibiciones supone situarse en escenarios de vulnerabilidad que también pueden tener repercusiones para las comadronas. Una respuesta a esta situación es que las mujeres acudan al centro de salud local sin el conocimiento de sus parejas, con el apoyo de las comadronas y enfermeras, que guardan las tarjetas de identificación de las mujeres para que sus maridos/parejas no puedan encontrarlas. Un análisis multinivel (individual, interpersonal, organizativo y nacional) revela que el acceso a la atención de salud depende no sólo de las infraestructuras, sino también de determinantes sociales y pautas culturales [20,25].

El éxito en la prevención y el control de las enfermedades de la iniciativa ETMI Plus requiere estrategias socioculturalmente apropiadas y sensibles a las problemáticas de género que impliquen a las partes interesadas locales y aborden la equidad étnica, de género y económica. No siempre es fácil determinar qué agentes esenciales deben participar en el proceso para que éste sea socioculturalmente apropiado y tenga en cuenta las problemáticas de género. La participación de múltiples actores, desde las comadronas hasta el nivel nacional de salud con el que mantenemos un estrecho diálogo a lo largo del diseño y la ejecución de este proyecto, ha tenido como objetivo producir un contenido coherente, respetuoso y culturalmente apropiado que debería tener aceptación a nivel comunitario, además de ser coherente con las directrices de la política nacional en torno a la ETMI Plus [18,20]. A través de la aplicación de **estrategias basadas en evidencia**, encontramos que la mayoría de las acciones en torno al diagnóstico y tratamiento ya estaban en marcha para las cuatro enfermedades; por lo tanto, nuestra propuesta se centra en el desarrollo de estrategias de material educativo y de comunicación que integran el VIH, la sífilis, la hepatitis B y la enfermedad de Chagas desde una perspectiva congénita [17]. Además, los retos que plantea la aplicación sistemática de una estrategia de pruebas homogénea pueden dar lugar a una aplicación desigual de las prácticas de salud, así como a los diferentes niveles de equipamiento de los distintos centros de salud. Las densidades de población desiguales, las vías de acceso y las múltiples condiciones sociales y geográficas que afectan a cada localidad determinan el número de personas que llegan a los centros de salud locales, así como sus características demográficas [45]. Es importante tener esto en cuenta a la hora de desarrollar programas de tamizaje y tratamiento para que consideren las particularidades de cada localidad. Por lo tanto, proponer el desarrollo de material educativo y de comunicación que integre las pruebas y los algoritmos de atención perinatal puede fortalecer los servicios actuales ofrecidos a las mujeres embarazadas. Hemos tomado como caso

Comapa para analizar sus características locales y cómo éstas demandan especial atención a la hora de diseñar intervenciones de salud.

Durante todo el desarrollo del proyecto se aplicaron **estrategias de participación** de la comunidad. Además, nuestro equipo había trabajado durante años anteriores aplicando estrategias de participación comunitaria centradas específicamente en la transmisión congénita de la enfermedad de Chagas [14,17]. Este modelo participativo sirvió de base para los esfuerzos de la estrategia para la ETMI Plus, ya que se habían establecido relaciones con el personal del Ministerio de Salud Pública y Asistencia Social, y otras instituciones gubernamentales y no gubernamentales relacionadas con la salud materno-infantil, incluidas las comadronas en el centro de los esfuerzos [25]. Aunque la participación de los distintos actores a través de un proceso consultativo ha sido un principio conceptual y metodológico fundamental de esta investigación, hemos tenido que sortear las múltiples luchas de poder y jerarquías presentes entre los distintos sistemas de conocimiento y otras tensiones sociales y culturales [10,34]. No es fácil transformar los patrones sociales y culturales, pero estamos de acuerdo con Hankivsky et al. (2010) en que, a través de la interseccionalidad y la investigación sensible al género, es posible anular las desigualdades estructurales mediante el desarrollo de métodos que diluciden el poder y tengan potencial para el cambio social [47].

El mapa de actores (Figura 4) sirvió para comprender la dinámica de poder que facilita o dificulta las funciones de cada actor. Aunque existe una ley nacional que regula las actividades de las comadronas, a nivel local ellas suelen encontrarse con múltiples obstáculos. Por ejemplo, desde la perspectiva del MSPAS, sólo se certifica oficialmente a las comadronas que asisten a un determinado número de talleres en el centro de salud local. Por lo tanto, surge otra capa en la dinámica de poder entre las comadronas “certificadas” y las “empíricas” [25]. Al haber trabajado sólo con las comadronas “certificadas”, hemos tenido que excluir las perspectivas de las matronas “empíricas” y de las mujeres a las que atienden.

La consulta constante con la comunidad, organizada a través del personal del centro de salud local, tiene la intención de facilitar y fomentar los canales de comunicación entre los distintos agentes de salud. Por lo tanto, si bien la centralización de los eventos (grupos focales, consultas y trabajo con las parteras acreditadas) puede implicar la reproducción de jerarquías de poder preexistentes, también pretende revertir estas jerarquías desde dentro a través de la propia metodología interseccional [10,14,25,34].

El trabajo con las comadronas también formaba parte de nuestras **estrategias socioculturales**, que pretendían identificar y considerar las particularidades del grupo objetivo. Aunque ha aumentado el número de mujeres embarazadas que acuden al centro de salud local para sus controles prenatales y el parto, todavía hay muchas mujeres que siguen buscando para la atención relacionada con el embarazo, a las comadronas de su comunidad o de comunidades vecinas. La preferencia por las comadronas puede

deberse al valor de sus conocimientos alternativos y su atención más personalizada, pero también puede estar relacionada con la falta de acceso a servicios de salud institucionales [16,45]. Por ejemplo, en algunas comunidades no hay carreteras adecuadas para llegar fácilmente al centro de salud local, y encontrar transporte puede tener un costo económico para las pacientes y sus familias. Las comadronas también suponen un costo para las familias, a diferencia del centro de salud local, que es gratuito. Esto implica que las valoraciones económicas influyen en las decisiones de las personas a la hora de buscar asistencia en salud.

Las comadronas llenan los vacíos que dejan sin cubrir las instituciones de salud públicas, que no siempre llegan a toda la población. Sin embargo, no pueden existir independientemente de ella. Por ejemplo, las comadronas no están capacitadas para tomar muestras de sangre, lo que forma parte de los objetivos de las estrategias para la ETMI Plus. Por lo tanto, las estrategias socioculturales desarrolladas entienden la complementariedad de los dos sistemas, en este caso, potenciando que las comadronas dirijan a las mujeres al centro de salud local para su atención perinatal y el parto, pero capacitándolas para que sean el primer puerto de escala para las preguntas sobre la ETMI Plus [14].

Una estrategia para asegurarlo ha sido el desarrollo de material educativo y de comunicación para ser utilizado por los diferentes actores (instituciones de salud pública, ONGs y comadronas) dirigido a diferentes audiencias: el personal de salud en su capacitación dirigida a las comadronas y las comadronas en su transferencia del mensaje a las mujeres embarazadas [14,17,20]. Teniendo en cuenta que las comadronas podrían tener un papel clave en su aplicación, el material debería incluir sus perspectivas socioculturales y sus conocimientos, y debería adaptarse a su nivel de alfabetización y a su idioma preferido. El uso de **estrategias lingüísticas y periféricas** nos ayudó a abordar estas cuestiones [17]. El desarrollo y la validación del material forman parte de la siguiente fase del proyecto, actualmente en curso.

Conclusión

Para concluir, esbozamos las contribuciones clave resultantes de un proyecto de investigación participativa que toma la interseccionalidad de género como herramienta metodológica y analítica central para mejorar el acceso a los servicios de tamizaje de salud materno-infantil.

En primer lugar, la integración de las perspectivas teóricas de la interseccionalidad y los determinantes sociales de la salud en una figura ilustrativa (Figura 1) ha pretendido aclarar la aplicación práctica de estas teorías en programas de adaptación cultural. En segundo lugar, el desarrollo de un mapa de actores relevantes (Figura 4) es transferible para la implementación de otras iniciativas de desarrollo comunitario, con ajustes específicos de contexto. Identificar a los actores locales, nacionales y

regionales importantes que deben coordinarse en la ejecución de programas de salud es una tarea compleja que sólo puede lograrse mediante el trabajo de campo, los compromisos a largo plazo sobre el terreno y el establecimiento de relaciones respetuosas con múltiples socios, idealmente de diversas disciplinas. Este proceso desentraña las estructuras del entorno de salud, pero debe tener en cuenta a las personas que ocupan los puestos, ya que estos cambios son susceptibles a cambios políticos que pueden influir en el predominio de unas estructuras sobre otras. En tercer lugar, la identificación de las vías de diagnóstico, prevención y tratamiento de las cuatro enfermedades de la iniciativa ETMI Plus (Figura 5) fue una contribución útil y potencialmente transformadora, ya que permite mejorar las futuras prácticas de salud de apoyo a la salud de mujeres y niños. El desarrollo de un protocolo unificado e integrado para diagnosticar y tratar estas enfermedades de transmisión vertical es el centro de proyectos en curso en la ONG asociada, y una investigación empírica como ésta, que produjo datos relevantes para el desarrollo de políticas públicas, es una contribución valiosa.

Por último, aunque no de forma exhaustiva, este proyecto ha pretendido demostrar una posible forma de aplicar programas de salud culturalmente adaptados, diseñados a través de una metodología participativa, sensible a las cuestiones de género, y destinados a la creación de productos concretos para uso y beneficio de la comunidad destinataria (material educativo y de comunicación para que las comadronas lo utilicen con las mujeres a las que atienden y para que el centro de salud local lo utilice en la formación de comadronas). Un propósito clave de estos materiales será también el cambio de las relaciones de poder dentro del sistema biomédico convencional, en el que las comadronas (y sus conocimientos alternativos) suelen quedar subsumidos bajo la jerarquía de la ciencia biomédica. En la próxima fase de desarrollo de los materiales y su uso piloto dentro de la comunidad a través de métodos participativos, esperamos involucrar a los diferentes actores en conversaciones que generen un espacio para el diálogo en una posición más igualitaria. Queda por ver si esto se puede conseguir. La importancia de este objetivo radica en que la propia metodología es un elemento clave en la consecución de los objetivos del proyecto y tiene una intención transformadora.

Lista de abreviaciones

ETMI Plus: Eliminación de la transmisión materno infantil del VIH, la sífilis, la hepatitis B y el Chagas; OPS: Organización Panamericana de la Salud; VIH: virus de la inmunodeficiencia humana; ETS: enfermedades de transmisión sexual; MSPAS: Ministerio de Salud Pública y Asistencia Social; DSS: determinantes sociales de la salud; COREQ: Criterios Consolidados para el Reporte de Estudios Cualitativos; USAC: Universidad de San Carlos de Guatemala; UVG: Universidad del Valle de Guatemala; DNDi: Iniciativa de Medicamentos para Enfermedades Desatendidas; COCODE: Concejos Comunitarios de Desarrollo; RENAP: Registro Nacional de Personas.

Declaraciones

Aprobación de ética y consentimiento de participación

El estudio obtuvo la aprobación ética del comité de ética de la Universidad del Valle de Guatemala (#196-05-2019). Se obtuvo el consentimiento por escrito de los participantes implicados y se eliminaron todos los identificadores personales por motivos de confidencialidad.

Consentimiento para publicación

No aplica

Accesibilidad a los datos y materiales

Los datos que utilizamos y/o analizamos para el presente estudio están disponibles previa solicitud razonable a través del autor correspondiente para respetar el acuerdo de confidencialidad del comité de ética que no permite la divulgación individual de los datos.

Conflicto de interés

Los autores declaran no tener ningún conflicto de interés.

Financiamiento

La Fundación Mundo Sano, una organización sin ánimo de lucro, financió la investigación y apoyó el diseño del estudio y la recolección de datos mediante un trabajo colaborativo. Todas las decisiones, incluidas las relativas a la publicación, se tomaron en colaboración entre todos los miembros del grupo de investigación.

Contribución de los autores

MRA participó en la conceptualización, metodología, análisis formal, investigación, recursos, curación de datos, redacción - borrador original, revisión y edición, supervisión, administración del proyecto y obtención de financiación del artículo. MG participó en la conceptualización, metodología, análisis formal, investigación, recursos, curación de datos, redacción - borrador original, revisión y edición, supervisión y obtención de financiación del artículo. EPR participó en la conceptualización, conservación de datos, redacción del borrador original, revisión y edición, visualización y obtención de fondos para el artículo. JGJ participó en los recursos, la curación de datos, la redacción - revisión y edición, y la visualización del artículo.

Agradecimientos

Nos gustaría dar las gracias a Ana Lucía Morales, Jorge Sincal, Roberto Chuit y Pamela Pennington por su apoyo en el trabajo de campo, la recolección de datos y la orientación conceptual. Agradecemos a Ángel del Valle sus valiosos comentarios sobre una primera versión del manuscrito.

References

1. PAHO/WHO. Chagas disease [Internet]. 2023 [citado el 4 de diciembre de 2023]. Recuperado a partir de: https://www.paho.org/en/topics/chagas-disease

2. Institute for Health Metrics and Evaluation (IHME). The Global Burden of Disease: Generating Evidence, Guiding Policy. Seattle, WA; 2013.

3. Bern C, Messenger LA, Whitman JD, Maguire JH. Chagas Disease in the United States: a Public Health Approach. Clin Microbiol Rev. 2019;33:e00023-19.

4. Antinori S, Galimberti L, Bianco R, Grande R, Galli M, Corbellino M. Chagas disease in Europe: A review for the internist in the globalized world. Eur J Intern Med. 2017;43:6–15.

5. Juarez JG, Pennington PM, Bryan JP, Klein RE, Beard CB, Berganza E, et al. A decade of vector control activities: Progress and limitations of Chagas disease prevention in a region of Guatemala with persistent Triatoma dimidiata infestation. PLoS Negl Trop Dis. 2018;12:17.

6. Panamerican Health Organization, World Health Organization. EMTCT PLUS. Framework for elimination of mother-to-child transmission of HIV, syphilis, hepatitis B and Chagas. 2017.

7. MSPAS. Plan Nacional para la Eliminación de la Transmisión Materno Infantil de VIH, sífilis y Hepatitis B 2021-2024. Guatemala; 2021.

8. Panamerican Health Organization. PAHO and Unitaid launch collaboration to advance the elimination of mother-to-child transmission of Chagas disease [Internet]. 2022 [citado el 25 de julio de 2022]. Recuperado a partir de: https://www.paho.org/en/news/30-6-2022-paho-and-unitaid-launch-collaboration-advance-elimination-mother-child-transmission

9. Proctor E, Silmere H, Raghavan R, Hovmand P, Aarons G, Bunger A, et al. Outcomes for Implementation Research: Conceptual Distinctions, Measurement Challenges, and Research Agenda. Adm Policy Ment Health Ment Health Serv Res. 2011;38:65–76.

10. World Health Organization. Community Engagement: A health promotion guide for universal health coverage in the hands of the people [Internet]. Geneva: World Health Organization; 2020 [citado el 14 de marzo de 2022]. Recuperado a partir de: https://www.who.int/publications/i/item/9789240010529

11. Política Nacional de Comadronas de los Cuatro Pueblos de Guatemala. 2015.

12. Cosminsky S. Midwives and Mothers: The Medicalization of Childbirth on a Guatemalan Plantation. 1st edition. University of Texas Press; 2016.

13. Organization World Health, editor. Incorporating intersectional gender analysis into research on infectious diseases of poverty: a toolkit for health researchers [Internet]. Geneva: World Health Organization; 2020 [citado el 19 de enero de 2022]. Recuperado a partir de: https://apps.who.int/iris/handle/10665/334355

14. Pennington PM, Juárez JG, Arrivillaga MR, Urioste-Stone SMD, Doktor K, Bryan JP, et al. Towards Chagas disease elimination: Neonatal screening for congenital transmission in rural communities. Chippaux J-P, editor. PLoS Negl Trop Dis. 2017;11:e0005783.

15. Social determinants of health [Internet]. [citado el 6 de marzo de 2023]. Recuperado a partir de: https://www.who.int/health-topics/social-determinants-of-health

16. Crear-Perry J, Correa-de-Araujo R, Lewis Johnson T, McLemore MR, Neilson E, Wallace M. Social and Structural Determinants of Health Inequities in Maternal Health. J Womens Health. 2021;30:230–5.

17. Kreuter MW, Lukwago SN, Bucholtz DC, Clark EM, Sanders-Thompson V. Achieving Cultural Appropriateness in Health Promotion Programs: Targeted and Tailored Approaches. Health Educ Behav. 2003;30:133–46.

18. Ghasemi E, Majdzadeh R, Rajabi F, Vedadhir A, Negarandeh R, Jamshidi E, et al. "Applying Intersectionality in designing and implementing health interventions: a scoping review". BMC Public Health. 2021;21:1407.

19. Torres-Ruiz M, Robinson-Ector K, Attinson D, Trotter J, Anise A, Clauser S. A Portfolio Analysis of Culturally Tailored Trials to Address Health and Healthcare Disparities. Int J Environ Res Public Health. 2018;15:1859.

20. Campbell KA, MacKinnon K, Dobbins M, Jack SM, for the British Columbia Healthy Connections Project. Nurse-Family Partnership and Geography: An Intersectional Perspective. Glob Qual Nurs Res. 2019;7:233339361990088.

21. Cho S, Crenshaw KW, McCall L. Toward a Field of Intersectionality Studies: Theory, Applications, and Praxis. Signs J Women Cult Soc. 2013;38:785–810.

22. Crenshaw K. Mapping the Margins: Intersectionality, Identity Politics, and Violence against Women of Color. Stanford Law Rev. 1991;43:1241.

23. Carbado DW, Crenshaw KW, Mays VM, Tomlinson B. INTERSECTIONALITY: Mapping the Movements of a Theory. Bois Rev Soc Sci Res Race. 2013;10:303–12.

24. Bowleg L. The Problem With the Phrase *Women and Minorities:* Intersectionality—an Important Theoretical Framework for Public Health. Am J Public Health. 2012;102:1267–73.

25. Holman D, Salway S, Bell A, Beach B, Adebajo A, Ali N, et al. Can intersectionality help with understanding and tackling health inequalities? Perspectives of professional stakeholders. Health Res Policy Syst. 2021;19:97.

26. West R, Bartkowski JP. Negotiating Patient-Provider Power Dynamics in Distinct Childbirth Settings: Insights from Black American Mothers. Societies. 2019;9:45.

27. Instituto Nacional de Estadística Guatemala. Resultados del XII Censo Nacional de Población y VII de Vivienda. 2019.

28. Hashimoto K, Cordon-Rosales C, Trampe R, Kawabata M. Impact of single and multiple residual sprayings of pyrethroid insecticides against Triatoma dimidiata (Reduviiade; Triatominae), the principal vector of Chagas disease in Jutiapa, Guatemala. Am J Trop Med Hyg. 2006;75:226–30.

29. Hashimoto K, Alvarez H, Nakagawa J, Juarez J, Monroy C, Cordón-Rosales C, et al. Vector control intervention towards interruption of transmission of Chagas disease by Rhodnius prolixus, main vector in Guatemala. Mem Inst Oswaldo Cruz. 2012;107:877–87.

30. Manne J, Nakagawa J, Yamagata Y, Goehler A, Brownstein JS, Castro MC. Triatomine Infestation in Guatemala: Spatial Assessment after Two Rounds of Vector Control. Am J Trop Med Hyg. 2012;86:446–54.

31. Monroy MC, Penados D, Pineda J, Ruiz EL, Agreda EO, Alcantara B, et al. A multidisciplinary, collaborative, inter-agency and comprehensive approach for the control of Chagas Disease as a public health problem in Guatemala. Acta Trop. 2022;225:106157.

32. Bernard HR. Research methods in anthropology: qualitative and quantitative approaches [Internet]. AltaMira Press; 2006. Recuperado a partir de: http://files/242/Russel-Research-Method-in-Anthropology.pdf

33. Creswell JW. Research Design Qualitative & Qualitative Approaches. Thousand Oaks, CA: SAGE Publications, Inc.; 1994.

34. De Urioste-Stone SM, Pennington PM, Pellecer E, Aguilar TM, Samayoa G, Perdomo HD, et al. Development of a community-based intervention for the control of Chagas disease based on peridomestic animal management: an eco-bio-social perspective. Trans R Soc Trop Med Hyg. 2015;109:159–67.

35. Tong A, Sainsbury P, Craig J. Consolidated criteria for reporting qualitative research (COREQ): a 32-item checklist for interviews and focus groups. Int J Qual Health Care. 2007;19:349–57.

36. Creswell JW. Qualitative inquiry and research design: Choosing among five approaches. 2nd edition. Thousand Oaks, CA: SAGE Publications, Inc.; 2007.

37. CARE Learning Tour. HEALTHY MOMS, HEALTHY SOCIETIES: Global Investments to End Maternal and Child Mortality in Guatemala. Guatemala: CARE Learning Tour; 2015.

38. Bossen LH. The Redivision of Labor: Women and Economic Choice in Four Guatemalan Communities. State University of New York Press; 1984.

39. Observatorio Regional de Planificación para el Desarrollo de América Latina y el Caribe, Sistema de Consejos de Desarrollo Gobierno de Guatemala. Consejos Comunitarios de Desarrollo Urbano y Rural (COCODE) de Guatemala | Observatorio Regional de Planificación para el Desarrollo [Internet]. 2009 [citado el 6 de marzo de 2023]. Recuperado a partir de: https://observatorioplanificacion.cepal.org/es/instituciones/consejos-comunitarios-de-desarrollo-urbano-y-rural-cocode-de-guatemala

40. López Ramírez R. Cosmovisión Xinca: una forma de humanizar la vida. Guatemala: Amanuense Editorial; 2007.

41. Berger-González M, Vides-Porras A, Strauss S, Heinrich M, Taquirá S, Krütli P. Relationships that Heal: Beyond the Patient-Healer Dyad in Mayan Therapy. Med Anthropol. 2016;35:353–67.

42. Karp I. Deconstructing culture-bound syndromes. Soc Sci Med. 1985;21:221–8.

43. Henderson S, Kendall E, See L. The effectiveness of culturally appropriate interventions to manage or prevent chronic disease in culturally and linguistically diverse communities: a systematic literature review. Health Soc Care Community. 2011;19:225–49.

44. Dean HD, Williams KM, Fenton KA. From Theory to Action: Applying Social Determinants of Health to Public Health Practice. Public Health Rep. 2013;128:1–4.

45. Stevens NR, Heath NM, Lillis TA, McMinn K, Tirone V, Sha'ini M. Examining the effectiveness of a coordinated perinatal mental health care model using an intersectional-feminist perspective. J Behav Med. 2018;41:627–40.

46. Sen G, Iyer A, Mukherjee C. A Methodology to Analyse the Intersections of Social Inequalities in Health. J Hum Dev Capab. 2009;10:397–415.

47. Hankivsky O, Reid C, Cormier R, Varcoe C, Clark N, Benoit C, et al. Exploring the promises of intersectionality for advancing women's health research. Int J Equity Health. 2010;9:5.
